# Generalized Vertical Components of built-up areas from global Digital Elevation Models by multi-scale linear regression modelling

**DOI:** 10.1371/journal.pone.0244478

**Published:** 2021-02-10

**Authors:** Martino Pesaresi, Christina Corbane, Chao Ren, Ng Edward

**Affiliations:** 1 European Commission, Joint Research Centre (JRC), Directorate for Space, Security & Migration, Ispra, Italy; 2 Faculty of Architecture, The University of Hong Kong, Pokfulam, Hong Kong SAR; 3 Institute of Future Cities, The Chinese University of Hong Kong, Hong Kong, Hong Kong SAR; Bristol University/Remote Sensing Solutions Inc., UNITED STATES

## Abstract

The estimation of the vertical components of built-up areas from free Digital Elevation Model (DEM) global data filtered by multi-scale convolutional, morphological and textural transforms are generalized at the spatial resolution of 250 meters using linear least-squares regression techniques. Six test cases were selected: Hong Kong, London, New York, San Francisco, Sao Paulo, and Toronto. Five global DEM and two DEM composites are evaluated in terms of 60 combinations of linear, morphological and textural filtering and different generalization techniques. Four generalized vertical components estimates of built-up areas are introduced: the Average Gross Building Height (AGBH), the Average Net Building Height (ANBH), the Standard Deviation of Gross Building Height (SGBH), and the Standard Deviation of Net Building Height (SNBH). The study shows that the best estimation of the *net* GVC of built-up areas given by the ANBH and SNBH, always contains a greater error than their corresponding *gross* GVC estimation given by the AGBH and SGBH, both in terms of mean and standard deviation. Among the sources evaluated in this study, the best DEM source for estimating the GVC of built-up areas with univariate linear regression techniques is a composite of the 1-arcsec Shuttle Radar Topography Mission (SRTM30) and the Advanced Land Observing Satellite (ALOS) World 3D–30 m (AW3D30) using the union operator (CMP_SRTM30-AW3D30_U). A multivariate linear model was developed using 16 satellite features extracted from the CMP_SRTM30-AW3D30_U enriched by other land cover sources, to estimate the gross GVC. A RMSE of 2.40 m and 3.25 m was obtained for the AGBH and the SGBH, respectively. A similar multivariate linear model was developed to estimate the net GVC. A RMSE of 6.63 m and 4.38 m was obtained for the ANBH and the SNBH, respectively. The main limiting factors on the use of the available global DEMs for estimating the GVC of built-up areas are two. First, the horizontal resolution of these sources (circa 30 and 90 meters) corresponds to a sampling size that is larger than the expected average horizontal size of built-up structures as detected from nadir-angle Earth Observation (EO) data, producing more reliable estimates for gross vertical components than for net vertical component of built-up areas. Second, post-production processing targeting Digital Terrain Model specifications may purposely filter out the information on the vertical component of built-up areas that are contained in the global DEMs. Under the limitations of the study presented here, these results show a potential for using global DEM sources in order to derive statistically generalized parameters describing the vertical characteristics of built-up areas, at the scale of 250x250 meters. However, estimates need to be evaluated in terms of the specific requirements of target applications such as spatial population modelling, urban morphology, climate studies and so on.

## 1) Introduction

### 1.1 Scope of the study

There is a world-wide established need and interest for information of built-up areas for various applications such as urban planning, urban climate studies, urban environment science investigation, city resilience and urban risk management [1]. In early 2000, the Shuttle Radar Topography Mission opened the era of Global Digital Elevation Models (DEM) processed automatically from remotely sensed data, and made available in the open scientific domain at a decametric spatial resolution [2, 3]. Nowadays, several global DEMs extracted from radar and optical sensors and targeting different aspects of Digital Surface Model (DSM) and/or Digital Terrain Model (DTM) specifications are available. Many of them are aligned with the Data Sharing Principles of the inter-ministerial Group of Earth Observation (GEO) and the Global Earth Observation System of Systems (GEOSS) [4] and constitute information that is potentially suitable for supporting decisions within, for example, the 2030 Agenda for Sustainable Development [5, 6].

This study aims to assess the possibility to use these free DEMs for extracting information on the vertical component of built-up areas. From the perspective proposed here, the purpose of the new information would be the spatial delineation and characterization of different parts of cities. Herein, *parts of cities* are to be interpreted as “areas morphologically homogenous” [7, 8] of the city taking into account the three-dimensional patterns of the buildings and the open spaces, in accordance with established urban morphology analysis methodologies [9–11]. The study sets a target spatial resolution of the extracted vertical information of sub-kilometric scale, allowing *city neighbouring analysis* [7–11]. The main use of this information would be to improve the open and free data available for systematic comparative studies of whole urban areas [12–14], global or regional urban sustainable development assessment [15], or spatial modelling exercises requiring the seamless coverage of the Earth’s surface as for instance the production of global population grid data by downscaling of census data [16]. On the other hand, it could be used to fill data gaps at the city or at regional level.

In this study, we extensively use the term “generalization” meaning the *statistical summary of spatial information collected at a more detailed scale and summarized to a less detailed scale*, in accordance with established spatial data analytics [17, 18] and spatial econometrics modelling [19] frameworks.

The study introduces the *GVC* of built-up areas as statistical summary at broad scale of some relevant geometric characteristics of the three-dimensional built-up environment collected at detailed scale. The first part of this study aims at understanding the available global DEM sources and select the best source to use as statistical summary, while the second part aims at analysing the estimates of GVCs of the built-up areas and proposing a final solution integrating the DEM sources with additional land cover information. The chosen data processing is based on well-established statistical methods, able to generalize the findings to the global context.

### 1.2 Relevance of built-up volumes

Information about the vertical components of the built-up areas are important in urban analysis, urban planning, and required in many studies in the built environment. The use of quantitative descriptions and prescriptions related to the three-dimensional size characteristics of the built-up structures (built-up volume) in relationships with the open spaces around them (plot, street), is a widely established approach in urban analysis and urban planning practices since mid of 19th century [20] and consolidated in urban morphology analysis practices [9–11]. Standard *urban density* measurements and management need an assessment of built-up volume in the building plot surface [21]. These basic parameters are needed to characterize the different planning and development zones and setting quantitatively the policy actions [22, 23]. At the single-building scale, the building footprint and the building height must be known in order to estimate the floor space, the floor space occupancy, crowding and other parameters such as energy requirements. The *urban density* calculated as built volume over the available plot surface [8, 10, 11] is a fundamental input variable for assessing the environmental sustainability of urbanization. According to the 5th Assessment Report (AR5) of the Intergovernmental Panel on Climate Change (IPCC), “key urban form drivers of energy and GHG emissions are *density*, land use mix, connectivity, and accessibility” [24]. Knowledge of the urban forms [11], in particular, the vertical dimension (i.e. building heights and volume) is necessary for a comprehensive characterization of urban morphology, especially for projecting future carbon emissions under scenarios of continuous urbanization and climate change [10, 25]. For example, the vertical dimension of urban areas is closely linked to the distribution of population densities [26] and may support urban climatology studies and related adaptation and mitigation actions [27–30]. The vertical dimension of built-up areas plays a major role in urban heat islands by affecting the urban energy balance [31], and is key in Earth System Modelling [32]. It can be used to understand the interlinkages between urban growth and vulnerability to natural hazards [16, 33–36]. Finally, urban forms are also essential to urban research with policy implications, such as urban planning and design, urban traffics and noise [37], air pollution dispersion [38], energy consumption [39], assessing solar potential [40, 41] and urban slums redevelopment [42].

### 1.3 Remote sensing of urban volume

Airborne digital photogrammetry combined with field surveys, with spatial resolution and error tolerances in the order of centimetres, target the description of single buildings in support to fine-scale cartography [43, 44]. This is the standard practice in urban analysis for local city administrations. Methods fusing data from airborne and spaceborne sensors, operating at the metric scale, target small clusters of buildings, and both direct and indirect methods have been developed for extracting three-dimensional information. Direct approaches consist in assessing building heights from Light Detection and Ranging (LiDAR) sensor data [45], stereoscopic satellite data [46] or aerial imagery, or through a fusion of LiDAR and optical or radar imagery [47, 48]. Indirect approaches use proxy variables to determine the volume of buildings such as building shadows [49, 50] or image derivatives and filtering of DSM. Global spaceborne scatterometer data has been investigated for characterizing urban growth and development in both the horizontal and vertical directions [51, 52] at the kilometric spatial resolution, targeting the description of entire urban agglomerations [53], or the internal characterization of large urban conurbations if used in in conjunction with other finer-scale information [54].

Some of these datasets are scarce and rarely available as open and free data because are typically costly. Consequently, they are rarely available in low-income countries and they are hard to use for globally-complete comparative studies. On the other hand, global spaceborne scatterometer data provide global consistency, but because of its limited spatial resolution, it cannot be used directly to delineate and characterize different parts of cities. This study explores the use of decametric spatial resolution spaceborne data for the morphological characterization of the different *parts of cities* [9–11], accordingly the vertical components of the buildings. The work in this domain was pioneered by Gamba et al. (1999) [55, 56], and conceptualized in the frame of global spatial data infrastructure supporting urban analysis by Nghiem et al. (2001) [57]. In more recent years, Geiss et al. (2019) [58], exploited the TanDem-X global DEM, with a spatial resolution of 0.4 arcseconds(∼12 m) to map building heights, and the same technology was recently integrated for continental-scale mapping of 3D building structure [59]. Quartulli and Datcu (2003) [60] and Gamba et al. (2012) [56] demonstrated the use of open and free SRTM global DEM for three-dimensional urban area characterization, despite its relatively coarse spatial resolution of 1 arcsecond (∼30 m) if compared to the buildings global average size of 10 m [61]. The use of these global DEMs for global systematic assessment of built-up areas was first experimented by Pesaresi et al. (2016) [62] in the context of the Global Human Settlement Layer (GHSL) that included a multiple-class urban land cover classification accounting for the built-up areas, vegetated surfaces, and vertical characteristics of built-up areas as inferred from the application of morphological and textural filtering of DEM data.

More recently, Wang et al.(2018) [63] and by Misra et al. (2018) [64] compared open global DEMs for estimating building heights either exclusively or in combination with multispectral data (e.g. Landsat imagery) [63]. Both studies highlighted the need for further investigations on the potential of global open DEMs to be used for mapping building heights and volumes under different terrain and urban density conditions.

### 1.4 Novelty of the study

The work presented here assesses the potential of open global DEMs in describing building height variation in different urban environments. Typically, the spatial resolution of Digital Building Height (DBH) model inherits the same spatial resolution of the input sensor data. In this study, a more abstract spatial modelling [17, 19] it is adopted by the introduction of an explicit spatial generalization processing step between the input DEM data and derived features at circa 30 m or 90 m of spatial resolution, and the built-up volumetric information estimated at the output spatial resolution of 250 m. This scale can approximate the spatial requirements for monitoring of global urban sustainable development [15], and specific global modelling exercises requiring information on building height and volume [16, 27, 29, 32, 65, 66]. DEM-derived spatial features extracted by convolutional, morphological and textural filtering techniques are generalized to the resolution of 250 m by statistical relations and the GVC of built-up areas are evaluated.

The study leverages on well-established methods and concepts. The novelty of the study relates to the specific combination of three main aspects: a) the strong open data and methods, b) the introduction of an abstract spatial generalization level where the model is evaluated, and c) the testing of new DEM spatial filtering techniques for the specific application.

Regarding the first aspect, the aim of the study is clearly set inside the best use of global baseline data publicly available in the full open and free access policy, which may have the potentiality to support the international development policies [6]. Moreover, the study is clearly set by the use of the lest-square linear regression that at the same time ensures well-established statistical structure, and increases the use of the model in real-world applications. Higher quality input data would have possibly increased model performances, but have been excluded from the study because not available in the global open free domain, that would consequently decrease scientific transparency and comparability or reproducibility of the results, thus finally decreasing the usability of the derived measures in the context of international policy frameworks. In turn, other inferential methods (e.g. machine learning) may have been adopted, possibly improving the model fitting to the tested data, but at the expense of computational efficiency, explainability of the findings, and potential loss of generality. Explainability and generality of the methods are keys for the usability of the derived measures in the context of public decision making processes, as the international development frameworks.

Regarding the second aspect, the resulting spatial resolution from the generalization introduced in the study is distinct from the spatial resolution of the input satellite data, while it is common practice to assume coincident input and output spatial resolutions. The size of the grids cell in output allows a coherent statistical evaluation of multi-scale spatial information extracted by heterogeneous spatial filtering from a heterogeneous set of input DEM data that are produced at different spatial resolutions.

Regarding the third aspect, while the spatial filtering techniques applied in the study are well-established, some specific concatenation of spatial filters tested in the study and their use in the context of spatially-generalized, predictive statistical models it is not common practice. Examples of novel spatial filtering tested in the study include the anisotropic, rotation-invariant texture measurements calculated from the composite of the morphological opening and closing residuals of the DEM data, the morphological gradient of the Laplacian convolutional filtering of the DEM data, and the morphological gradient of the composite of morphological opening and closing residuals of the DEM data. Moreover, image morphological filters are commonly applied in an apodictic-deductive formal reasoning, descending the necessary characteristic of the filters from the formally explicit characteristics of the target pattern to be detected in the data that must be exactly and completely known a priori before the analysis. In this study, a different approach was set inside a spatial generalization and statistical inductive reasoning frame.

## 2) Study area and data

### 2.1 Test cases and reference data

Six test cases were selected within six different cities: Hong Kong, London, New York, San Francisco, Sao Paulo, and Toronto. The selection of the sites was mainly driven by the availability of cartography in digital format including information on building heights between 2016 and 2018. The selection aims at maximizing, given the limited available data, the heterogeneity of urban morphologies. Prior to their inclusion, the sources were revised by visual inspection and comparison with sub-meter optical satellite data in order to assess: i) reliability, ii) validity, and iii) the date of last update.

All vector datasets were rasterized to the 1 m spatial resolution reporting the information about the height of buildings in a DBH reference model. This constitutes the input used to generate the reference GVC of built-up areas at the spatial resolution of 250 m. For the six cities, a total of 74744 grid cells (samples) of 250 m spatial resolution were obtained. Some of them where at the edge of the valid spatial domain, thus meaning that a share of non-valid data points were to be incorporated in the generalization. A selection of samples from strictly valid data produced a subset of **59566** samples that were effectively used in this study. A summary of the characteristics of the reference datasets per study area is given in Table 1.

**Table 1:** Characteristics of the study areas: The mean height of buildings is calculated as surface-weighted average of the buildings heights digitized at 1 m resolution.

	HONG KONG	LONDON	NEW YORK	SAN FRANCISCO	SAO PAULO	TORONTO
**No. of samples**	13802	24908	9247	1600	15204	9896
**No. of valid samples**	5962	20498	8532	1600	13748	9226
**No. of buildings**	683325	649453	1082751	173878	2624665	420720
**Area of buildings (km^2^)**	97.18	209.87	159.59	32.00	291.79	114.52
**Total surface of the study area (km^2^)**	1099.75	1590.44	783.13	125.07	997.65	641.69
**Maximum height of buildings (m)**	485	285	472	286	319	290
**Mean height of buildings (m)**	18.8	13.5	15.0	14.5	6.4	14.1
**Date**	2016	2016	2018	2018	2018	2018

### 2.2 Digital Elevation Models

Seven different global DEMs, including two DEM composites, are evaluated in this study. Two versions from the Shuttle Radar Topography Mission at 1 arcsec (SRTM30) and 3 arcsec (SRTM90) of spatial resolution [3] [67–69], the Advanced Spaceborne Thermal Emission and Reflection Radiometer (ASTER) Global Digital Elevation Model available at ~30 m of spatial resolution [70–72], the Advanced Land Observing Satellite World 3D (AW3D30) available at ~30 m of spatial resolution [73], and the Multi-Error-Removed Improved-Terrain (MERIT) DEM available at ~90 m resolution [74]. More technical details on these DEMs are available in the S1 File, and their technical features and limitations are discussed in [75]. The two composite DEMs are derived from the AW3D30 and SRTM30 by using the point-wise extrema operators maxima and minima, producing the “CMP_SRTM30-AW3D30_U” and “CMP_SRTM30-AW3D30_I”, respectively.

## 3. Methodology

### 3.1 Experimental setting

**Fig 1:**
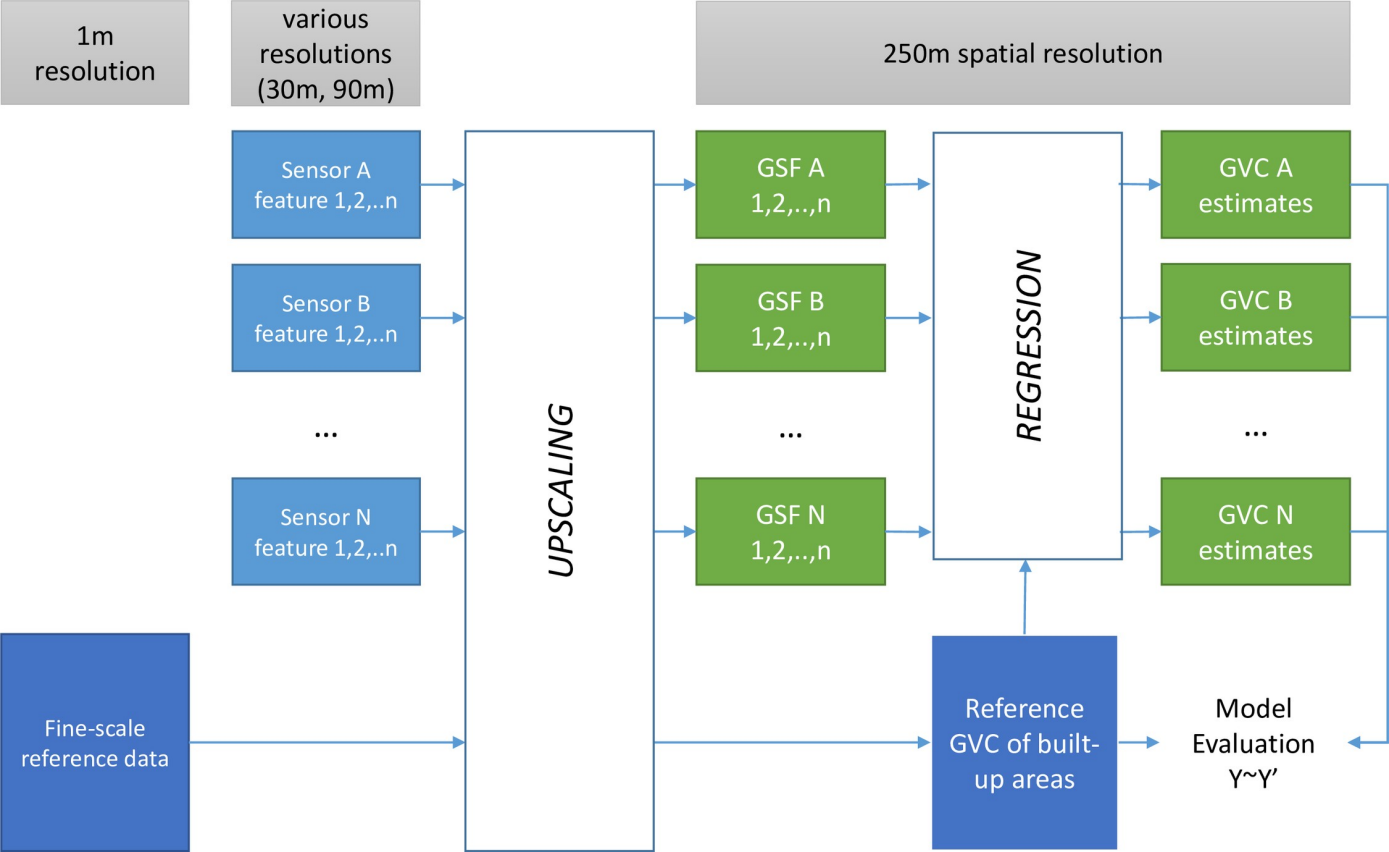
General logic schema of the study.

The general workflow of the study is shown in Fig 1. Various Generalized Satellite Features (GSF) are derived at 250 m resolution from DEM and satellite data input at circa 30 m and 90 m native spatial resolution. A set of vertical components of built-up areas are digitized from reference cartography at 1 m spatial resolution and used as input to generate the GVC of built-up areas at 250 m resolution; namely the AGBH, ANBH, SGBH, and SNBH. Average and standard deviation operators are used in the upscaling generalization process. The different GSF extracted from the DEM data and other satellite data are evaluated in this study trough linear regression techniques, that are used to infer GVC of built-up areas from the GSFs used as independent variables.

### 3.2 The Generalized Vertical Components (GVC) of built-up areas

The GVC of built-up areas summarize at broad scale some relevant geometric characteristics of the three-dimensional built-up environment collected at a more detailed scale. Let be *X* a given raster sample grid at 1 m of spatial resolution, and *x_i_* the measure of the building height reported at each position of *X*. Be *N* a generalized raster grid having the same origin of the grid *X*, and the cell of size 250 m. Be *n* the number of *x_i_* spatial samples that are included in the *N* grid cell, with *n* = 250^2^ = 62500.

Four GVC of built-up areas aggregating the information of *X* to the larger neighboring *N* are defined: they are the Average Gross Building Height (AGBH), the Average Net Building Height (ANBH), the Standard Deviation of Gross Building Height (SGBH), and the Standard Deviation of Net Building Height (SNBH).

$$AGBH=\left\{\frac{1}{n}\sum_{i=1}^{n}x_{i}\;\forall \;x_{i}\in N\right\}$$ (Eq 1)

$$ANBH=\left\{\frac{1}{n}\sum_{i=1}^{n}x_{i}\;\forall \;x_{i}\in N|x_{i}>0\right\}$$ (Eq 2)

$$SGBH=\left\{\sqrt{\frac{1}{n-1}\sum_{i=1}^{n}{\left(x_{i}-\bar{x}\right)}^{2}}\;\forall \;x_{i}\in N\right\}$$ (Eq 3)

$$SNBH=\left\{\sqrt{\frac{1}{n-1}\sum_{i=1}^{n}{\left(x_{i}-\bar{x}\right)}^{2}}\;\forall \;x_{i}\in N|x_{i}>0\right\}$$ (Eq 4)

**Fig 2:**
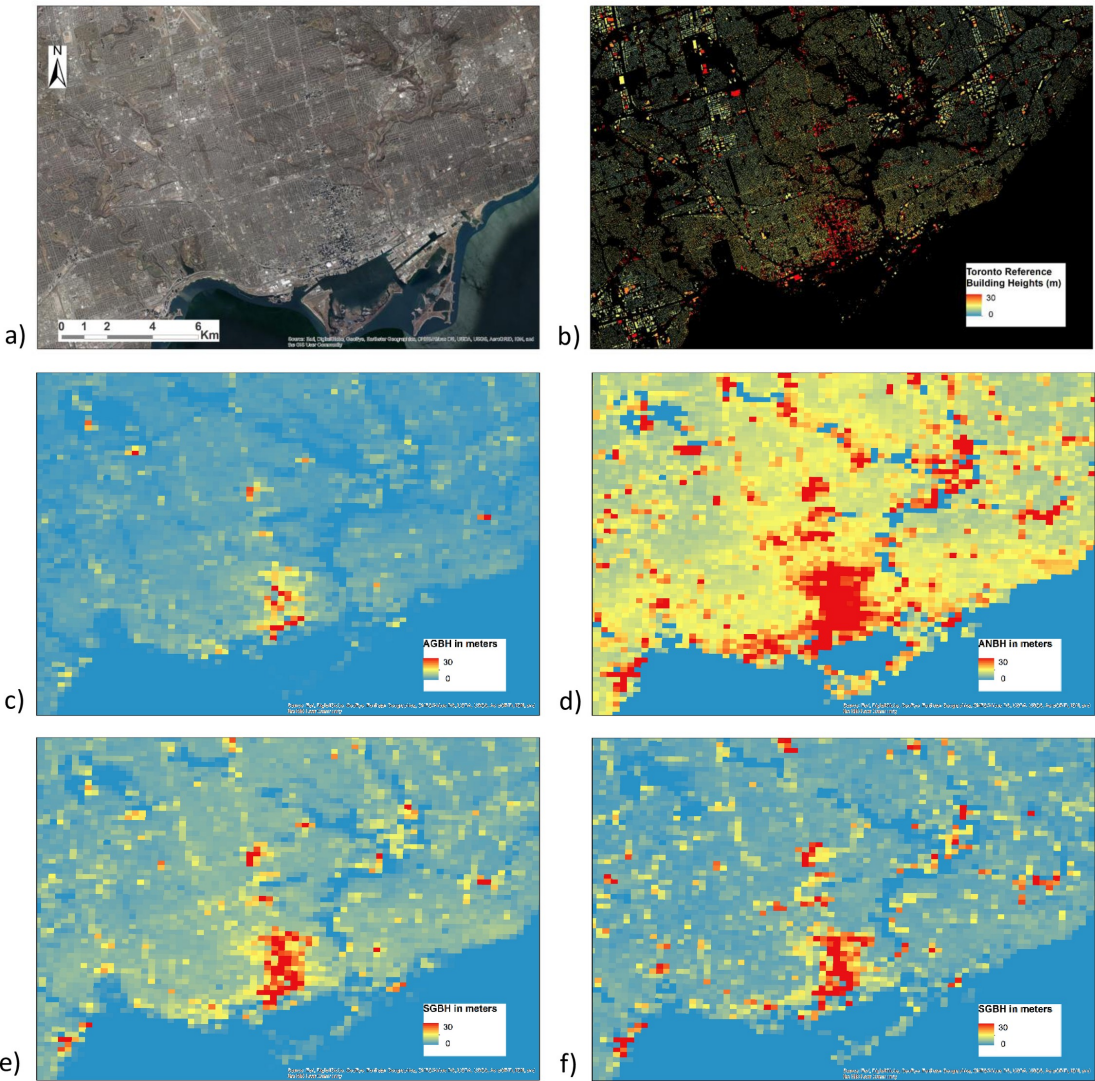
250 m A sample of the input reference data for the city of Toronto and the derived GVC of built-up areas. a) Very high resolution satellite data, b) the 1 m reference grid on building footprints and their heights (data source: City of Toronto Open Data Portal https://open.toronto.ca/), and the 250 m grids GVCs c) AGBH, d) ANBH, e) SGBH, and f) SNBH (image sources: Esri, DigitalGlobe, GeoEye, i-cubed, USDA FSA, USGS, AEX, Getmapping, Aerogrid, IGN, IGP).

In the “gross generalization” (AGBH, SGBH) all the fine grid cells inside a coarser cell are used in the calculation, while in the “*net generalization*” (ANBH, SNBH) only the fine grid cells with a support value greater than zero are considered in the statistical summary reported at the coarser grid cell. It is worth noting that by definition the net generalization is more sensitive to the scale or detail of the input satellite data than the gross generalization. Fig 2 shows an example of the GVCs corresponding to the city of Toronto as extracted from reference digital cartography.

Gross and net generalization are complementary and they may be relevant for distinct spatial modelling applications. Typically, the gross generalization can be used for spatial downscaling; for instance, of census population data [16, 76]. Or to estimate parameters necessary for delineation of local Urban Climate Zones [27–29, 65]. On the other hand, the net generalization could support modeling requiring precise parametrization of indicators extracted at the scale of the single building. Examples of the latter include indicators on floor surface occupancy [22], analysis of housing conditions [77] and slum assessment [78], or energy requirements and balance [39, 79].

From the GVCs as defined above, some measures commonly used in urban analysis can be derived as detailed in the S1 File.

By definition of the AGBH and ANBH, the net built-up surface share in the N grid cell can be derived from the AGBH and ANBH as: $\frac{\sigma_{BU}}{\sigma_{Unit}}=\frac{AGBH}{ANBH}$. With *σ_BU_* the total net built-up surface of the buildings included in the grid cell N, and *σ_Unit_* the total surface of the same grid cell N.

Furthermore, assuming a uniform ceiling height *h* of the housing units in the built-up structures summarized at the given spatial unit *N*, the total *floor surface σ_Floor_ m*^*2*^ in the same spatial unit *N* can be derived from the AGBH and ANBH as: $\sigma_{Floor}=\frac{AGBH∙\sigma_{Unit}}{h}$ and $\sigma_{Floor}=\frac{{ANBH}^{}∙\sigma_{BU}}{h}$, correspondingly.

**Fig 3:**
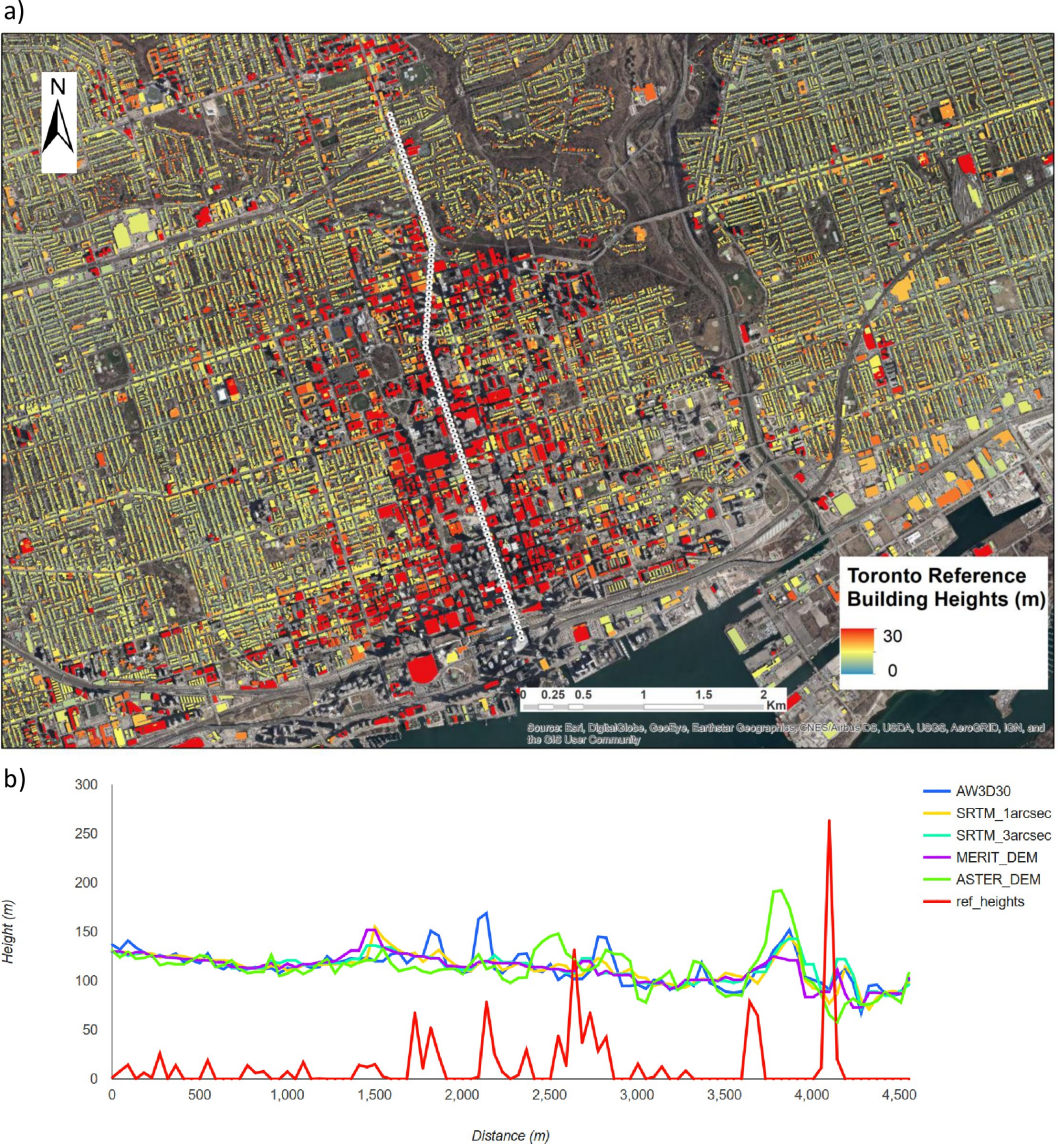
Height profiles calculated for a transect crossing the city centre of Toronto from five different DEMs. a) Detail from the 1 m reference grid on building footprints (data source: City of Toronto Open Data Portal https://open.toronto.ca/), b) DEM height profiles at their native spatial resolution (image sources: Esri, DigitalGlobe, GeoEye, i-cubed, USDA FSA, USGS, AEX, Getmapping, Aerogrid, IGN, IGP).

Fig 3 shows an example of building heights profiles calculated for a transect (white polyline) crossing the city centre, the residential area and a forest area in Toronto. The height profiles are calculated from the reference building footprints and the five difference elevation datasets in their native resolutions.

### 3.3 Generalized Satellite Features (GSF)

#### 3.3.1 GSF from DEM data

Thirty different features (see Table 2) are extracted from the DEMs by using well-established convolution, morphological, and textural spatial filtering methods based on the processing of the spatial neighbouring information. Spatial filtering was applied in a multi-scale approach: a set of alternatives regarding the size of the spatial neighbouring applied in the filter was tested. All the applied spatial filters have been designed to extract or emphasize the high-spatial-frequency information presumably related to variations associated to the presence of built-up structures, as compared to lower-spatial-frequency information related to the terrain.

**Table 2:** The list of features derived from spatial filtering of the input DEM data.

ID	DEM filter	Description
1	f1	f1 Laplacian linear convolution filter 3x3—central kernel weight = 8
2	f2	f2 Laplacian linear convolution filter 3x3—central kernel weight = 9
3	f3	f3 Laplacian linear convolution filter 3x3—central kernel weight = 10
4	f1d1	Morphological Derivative (Dilation—Erosion) of f1, Structuring element = 3x3
5	f1d2	Morphological Derivative (Dilation—Erosion) of f1, Structuring element = 5x5
6	f1d3	Morphological Derivative (Dilation—Erosion) of f1, Structuring element = 11x11
**7**	f2d1	Morphological Derivative (Dilation—Erosion) of f2, Structuring element = 3x3
8	f2d2	Morphological Derivative (Dilation—Erosion) of f2, Structuring element = 5x5
9	f2d3	Morphological Derivative (Dilation—Erosion) of f2, Structuring element = 11x11
**10**	f3d1	Morphological Derivative (Dilation—Erosion) of f3, Structuring element = 3x3
11	f3d2	Morphological Derivative (Dilation—Erosion) of f3, Structuring element = 5x5
12	f3d3	Morphological Derivative (Dilation—Erosion) of f3, Structuring element = 11x11
13	m1	m1 morphological transform: opening residuals—closing residuals, Structuring element = 3x3
14	m2	m2 morphological transform: opening residuals—closing residuals, Structuring element = 5x5
15	m3	m3 morphological transform: opening residuals—closing residuals, Structuring element = 11x11
16	m1d1	Morphological Derivative (Dilation—Erosion) of m1, Structuring element = 3x3
17	m1d2	Morphological Derivative (Dilation—Erosion) of m1, Structuring element = 5x5
18	m1d3	Morphological Derivative (Dilation—Erosion) of m1, Structuring element = 11x11
**19**	m2d1	Morphological Derivative (Dilation—Erosion) of m2, Structuring element = 3x3
**20**	m2d2	Morphological Derivative (Dilation—Erosion) of m2, Structuring element = 5x5
21	m2d3	Morphological Derivative (Dilation—Erosion) of m2, Structuring element = 11x11
22	m3d1	Morphological Derivative (Dilation—Erosion) of m3, Structuring element = 3x3
23	m3d2	Morphological Derivative (Dilation—Erosion) of m3, Structuring element = 5x5
24	m3d3	Morphological Derivative (Dilation—Erosion) of m3, Structuring element = 11x11
25	ptxiw3	GLCM contrast textural measurement 8directions of m1, anisotropical intersection wsize = 3x3
26	ptxiw5	GLCM contrast textural measurement 8directions of m1, anisotropical intersection wsize = 5x5
27	ptxiw11	GLCM contrast textural measurement 8directions of m1, anisotropical intersection wsize = 11x11
28	ptxuw3	GLCM contrast textural measurement 8directions of m1, anisotropical union wsize = 3x3
29	ptxuw5	GLCM contrast textural measurement 8directions of m1, anisotropical union wsize = 5x5
30	ptxuw11	GLCM contrast textural measurement 8directions of m1, anisotropical union wsize = 11x11

All spatial filters are computed in the DEM native grid geometry (spatial resolution, global projection, grid origin). Textural measurements are based on the Grey Level Co-occurrence Matrix (GLCM).

Convolution filters are additive filters: they are computed by adding each element of the DEM raster data to its local neighbours, weighted by the kernel values [80]. In particular, a specific class of convolutional filtering named “Laplacian” that are approximating the first discrete derivative of the input signal are used in this study for sharpening the DEM data [81]. In their original formulation of Serra (1983) [82] image Morphological filters are non-additive: they are computed by non-linear operators such a min/max extrema applied in the systematic comparison between the input raster function and a given target raster *structuring element* (SE), being the SE a mathematic operator including specific size, shape and symmetry characteristics translated in the raster discrete grid. Morphological filters are typically used for non-linear de-noising and spatial band-pass or shape granulometry purposes [83]. The Morphological filters applied in this study are based on the union of opening and closing residuals, on the morphological gradient calculated as the difference between the dilation and the erosion, and on the chaining of these two morphological transforms in order to estimate first and second-order discrete derivative. More details on these filters are available in the S1 File.

The textural filtering used in this study are derived from the work of Haralick (1973) [84] extracting statistical descriptors of recurrent spatial patterns in raster data from the observation of the Grey Level Co-occurrence Matrices (GLCM), governed by a *displacement vector* parameter setting the spatial pattern under test, and a *window size* parameter setting the scale of the derived textural measures. Coherently with the general approach of the study, the applied textural filters are all based on the Contrast GLCM measurement with a displacement vector length of 1 pixel and eight directions, extracting high-spatial-frequency contrast information. The textural parameters that are tested in this study are two: i) the intersection vs. union operator (respectively min vs. max point-wise transform) composing the anisotropic contrast measurements from different directions and ii) the window size setting the scale of the extracted textural information.

More details on the mathematical formulation of the DEM-derived GSF are included in the S1 File.

The GSF are extracted from the generalization of the DEM spatial filtering done at the DEM native resolution and global projection. During the generalization step, the GSF raster grids are standardized in resolution (250 m) and projection correspondingly to the local UTM projection. The generalization step is supported by two statistical operators summarizing the DEM-filtered values from the native raster grid at ~30 m or ~90 m spatial resolution to the 250 m grid cell: namely, by average and by standard deviation. Thirty DEM spatial filters were tested at the DEM native raster geometry: consequently, (30x2) 60 different GSFs are computed and evaluated in this study as independent variables at 250 m spatial resolution. The complete list of the 30 DEM spatial filters applied in this study before the generalization step is provided in Table 2, with a short mnemonic description.

#### 3.3.2. Ancillary GSF

The presence of buildings and the presence of vegetation canopy are known factors influencing the characteristics of the DEMs [71–73]. In this study, those factors are tested as additional GSF extracted from non-DEM data also available globally in the open and free domain. The presence of buildings was approximated by the share of built-up surface in the 250 m spatial resolution as resulting by aggregating the 30 m-resolution “built-up” land cover data generated by the GHSL [12], that, according to [12], is a good estimator (R^2^ = 0.89) of the sum of built-up surface. The GHSL built-up area data includes multi-temporal information organized in four years (1975, 1990, 2000 and 2014). The built-up grid for year 2014 was preferred since it was derived from Landsat data acquisitions from 2012-01-01 to 2014-12-30 and considered as the closest to the temporal range of the DEMs (2000–2011) included in this study.

Together with the presence of elevated built-up structures, vegetation plays a significant role in affecting the quality of DEMs in terms of vertical noise. In this study, the characterization of vegetation relies on the Normalized Difference Vegetation Index (NDVI), assessed from Landsat images matching the dates of the built-up areas. The pixel with the highest value of NDVI (i.e. greenest pixel) was considered as the composite value, in order to normalize the seasonal vegetation changes. More details on assessing greenness in urban areas are given in [85]. Greenness values were classified into three classes:

- “veg1” class—Low green for greenness < 0.1: corresponding to barren rocks, sand, snow or impervious surfaces (e.g. built-up areas)
- “veg2” class—Medium green for 0.2 <Greenness <0.5: corresponding to shrubs or agriculture
- “veg3” class—High green for 0.6 <Greenness < 0.9: corresponding to dense vegetation (e.g. forest, private gardens, etc.).

The combination of the three vegetation intensity classes {veg1, veg2, veg3} with the two classes expressing the presence vs. the absence of built-up surface {bu, nbu} generates six classes named “veg1bu”, “veg2bu”, “veg3bu”, “veg1nbu”, “veg2nbu”, “veg3nbu”. These classes are generated from input raster data at 30 m of spatial resolution and successively they are generalized to the 250 m spatial resolution grid by the application of the mean operator to each single class taken individually.

**Fig 4:**
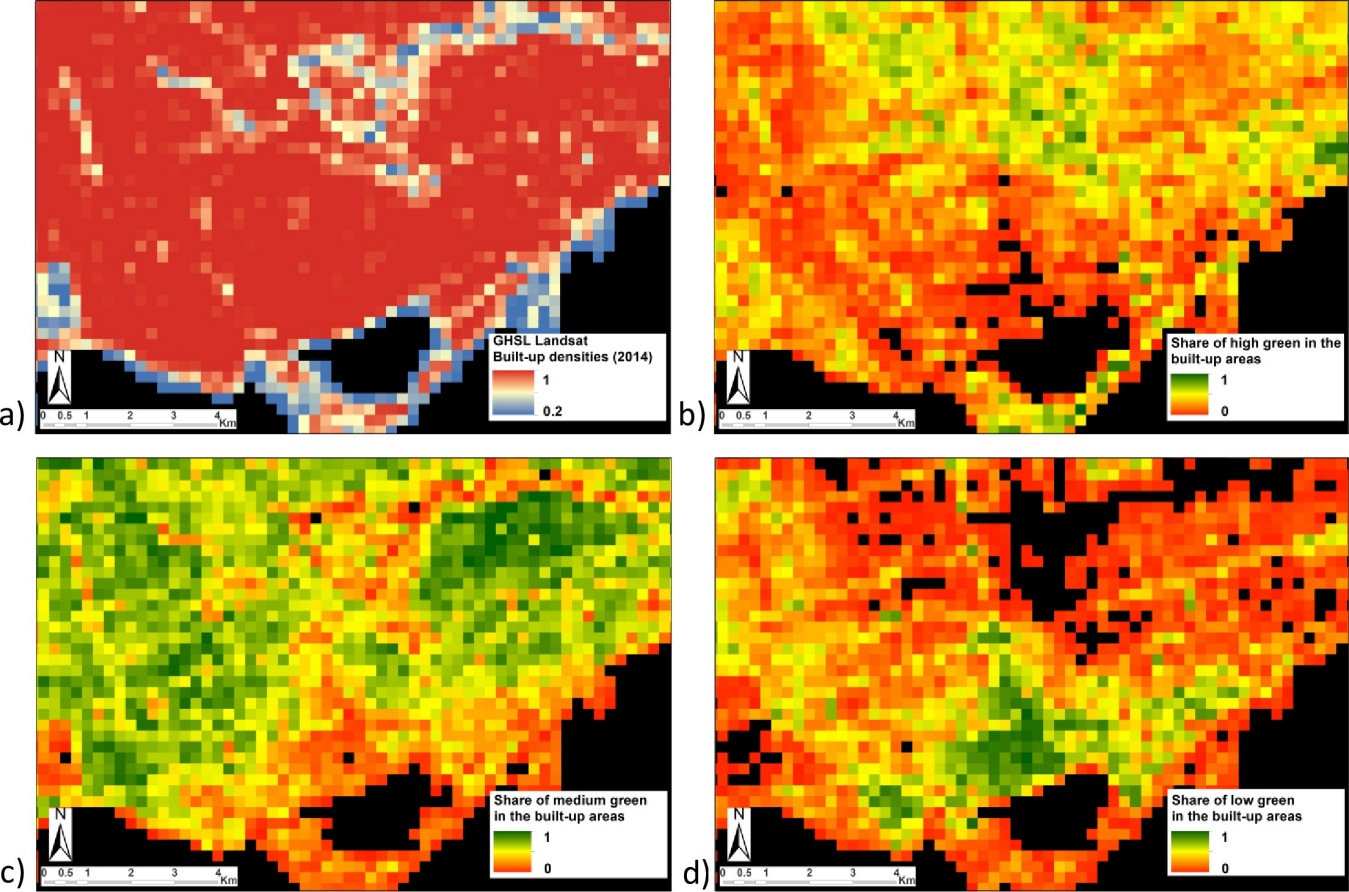
Illustration of the ancillary features characterizing built-up areas for the city of Toronto. a) Built-up densities, b) high green surfaces (veg3bu), c) medium green surfaces (veg2bu) and d) low green surfaces (veg1bu).

An illustration of the different ancillary features used for describing the built-up areas is shown in Fig 4, relatively to the city of Toronto.

### 3.4 Regression analysis

#### 3.4.1 Univariate regression analysis

Each GSF extracted by each global DEM and DEM composite, was tested individually as estimator of GVC of built-up areas using least-square linear regression. Performance is assessed by the RMSE of the estimated vs. the observed GVC of built-up areas. The use of a first-degree polynomial function in the least-square regression is justified by the need of generality and simplicity. Univariate regressions were tested for each city separately and for all the cities together (7 test cases). In total, (60 GSF) X (7 DEM) X (4 GVC) X (7 TEST CASES) = 11760 combinations were tested.

#### 3.4.2 Multivariate linear regression (MLR) analysis

A Multiple Linear Regression (MLR) approach considering the whole set of valid samples (59566 across the six test cases) of 250 m resolution was adopted, in coherence with the need of generality and simplicity driving the overall study. Statistical analysis revealing individual p-values allows discriminating between useful and less useful GSF predictors individually. Multicollinearity has clear implications for the size and the standard error of the regression coefficients [86] and it weakens the statistical power of the regression model. Therefore, pairwise correlation coefficients (*r*) were computed for all the 67 GSFs. If two GSFs have a high correlation, the one with the largest mean absolute correlation coefficient is dropped from the MLR. In order to understand the contribution of the GSFs to the prediction of specific GVCs, an analysis of relative importance is performed following the method of variance decomposition based on averaging over orderings proposed by Lindeman, Merenda and Gold and referred here as LMG metric [87]. The LMG metric decomposes the squared correlation *R*^2^ into non-negative contributions that sum to the total *R*^2^ [88]. Despite being computationally heavy especially in the presence of a large number of predictors, the LMG metric has been largely recommended for determining a ranking of the predictors and for quantifying their respective importance in the regression model [89].

From the original list of 67 GSF variables assessed in the study, a subset of 16 GSF variables to be included in the model was done by collinearity analysis, statistical significance assessment, and the assessment of the relative importance of model predictors by application of the LMG metric. This subset of variables was tested under the hypothesis that a smaller set of GSF variables may lead to better performing models. This was done by a stepwise selection technique based on sequential replacement that is a combination of forward and backward selections [90]. The process starts with no predictors, and then sequentially adds the most contributing predictors (like forward selection). After adding each new variable, the process removes any variables that no longer provide an improvement in the model fit (like backward selection). In the process, the Akaike Information Criterion (AIC) [91] was used to compare the alternative model performances and decide between model candidates.

### 3.5 Model performances assessment

In the univariate regression, the performance of the model is assessed by observation of the Root Mean Square Error (RMSE) of the predicted GVC vs. the observed GVC of built-up areas. The RMSE it is widely accepted metric to assess the amount of absolute error of models, and it is expressed in the same units of the dependent variables (meters in this study). The RMSE was calculated for the whole set of valid data (n = **59566** samples) labelled as test case “All”. Single test case RMSE was obtained by calibrating the univariate model using the data of the tests cases taken separately.

In the multivariate regression, the quality of models fit was assessed using the RMSE and the adjusted coefficient of determination (adj.R^2^). The MLR techniques adopted in this study are based on the search of the best regression coefficients minimizing the quadratic error. In this work, we calculated two different versions of the RMSE: i) the RMSE of the full set of observations (n = **59566** samples) and ii) the RMSE from the repeated k-fold cross-validation to estimate the prediction error rate (RMSE_CV). The repeated K-fold cross-validation is considered a robust method for estimating the accuracy of the models and assessing its generalization capabilities. The number of folds was set to 5 and the repetitions to 3. These values have shown empirically to yield test error rate estimates that suffer neither from excessively high bias nor from very high variance [92]. The adj.R^2^ is a widely accepted metric for assessing the amount of relative error of models. It is a statistical measure expressed in the interval [0 1] that shows the proportion of variation explained by the estimated regression line, by mitigating the risk of inflation of R^2^ when extra explanatory variables are added to the model [93].

## 4) Results

### 4.1 Univariate regression analysis

**Fig 5:**
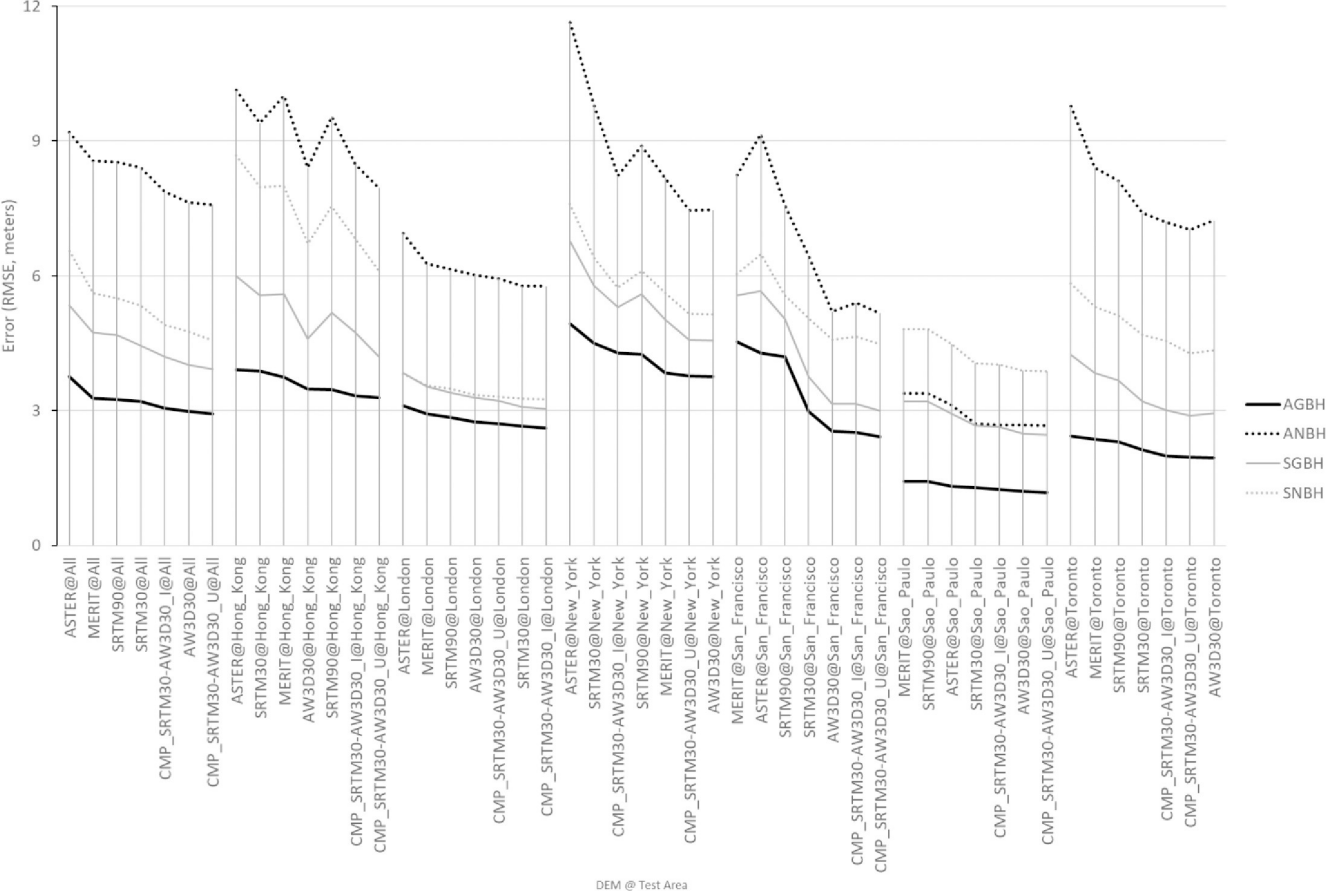
Minimal RMSE in the prediction of the GVCGVC of built-up areas, across 60 different input GSFs extracted from global DEM data, by DEM source and test area. The test area name “All” on the left of the chart reports about the RMSE obtained by estimating the regression parameters using the total number of valid samples (59566) considered in the study.

Fig 5 shows the best prediction (*minimal error across all the 60 considered GSFs*) of the four GVCs, by DEM or DEM composite source and by study area. In the chart, the results are ranked by decreasing error of the AGBH estimates. The best estimation was obtained for the AGBH, showing a RMSE < 3m for the all the case studies. Independently from the DEM and the test areas, the best estimation of the net vertical characteristics of built-up areas (ANBH, SNBH), always shows greater error than their corresponding gross estimation (AGBH, SGBH), both in terms of mean and standard deviation. The correlations between the all DEM-derived GSFs and the four GVCs as obtained during the univariate regression analysis are displayed in the S1 File for each considered input DEM.

Looking at the performances of the different DEM sources independently from the test areas, the least performing DEMs are systematically ASTER and MERIT, while the most performing are the AW3D30, the SRTM30, and the composite CMP_SRTM30-AW3D30_U. In the context of this study, the limiting factors influencing the DEM performances are most likely a) the spatial resolution of the DEM, b) the characteristics of the satellite data used to generate the DEM (radar vs. optical sensor technology, spatial resolution), and c) the applied DEM processing and post–processing chain including filtering and gap-filling steps.

The second and third limiting factors are possibly explaining the relative low performances of ASTER and MERIT DEMs. In particular regarding ASTER, the main limiting factor is possibly the characteristics of the satellite data (from optical sensor at 15m spatial resolution) unable to resolve the average size of the built-up structures [70]. Regarding MERIT, the relative low performances in estimating the GVC of built-up areas are possibly explained by the filtering out of the signal related to built-up areas considered as noise in respect to the bare earth [74].

**Fig 6:**
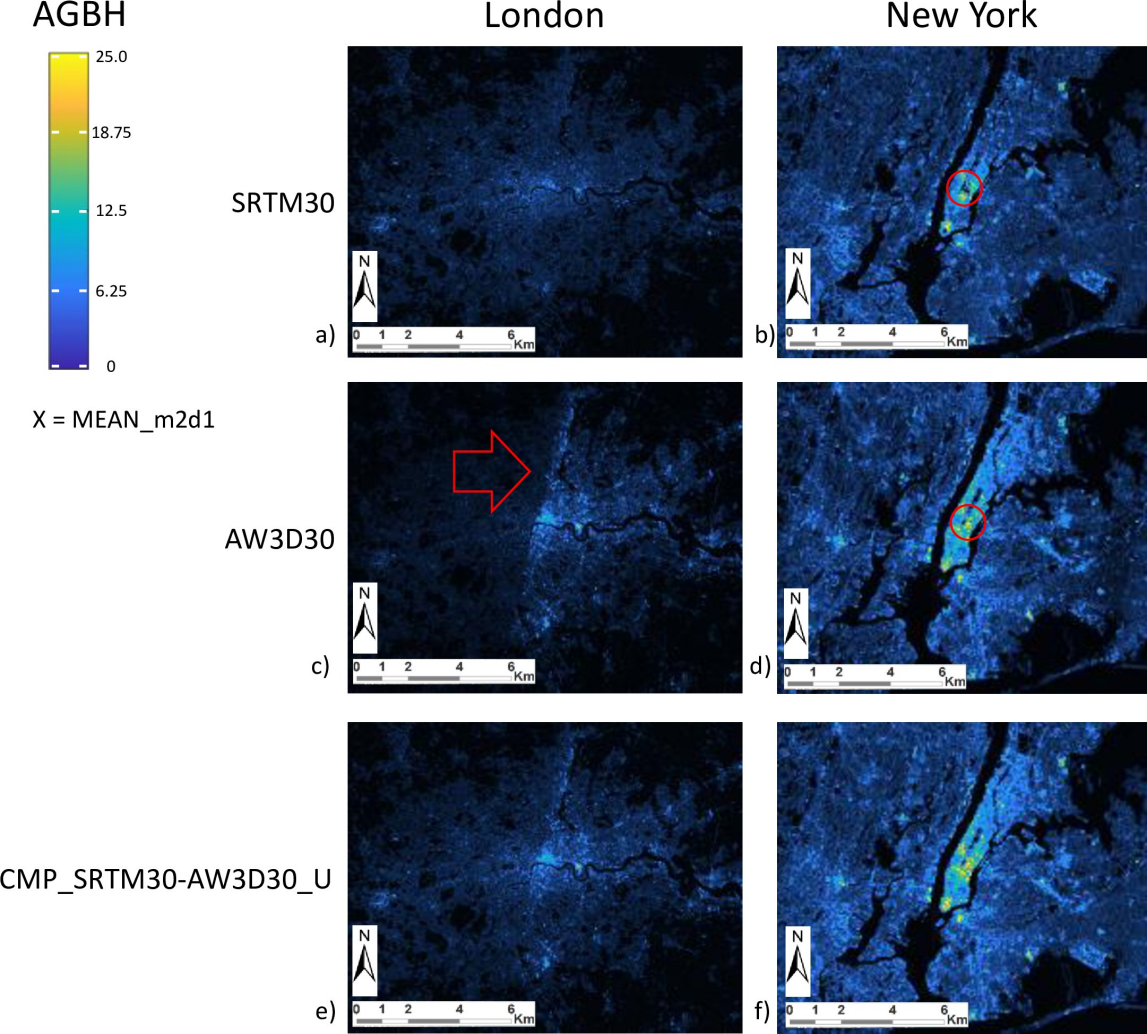
Example of AGBH as estimated by univariate regression using the MEAN_m2d1 DSF extracted from alternative DEMs in the two test cases of London (a,c,e) and New York (b,d,f). From top to bottom: SRTM30 (a,b), AW3D30 (c,d), and CMP_SRTM30-AW3D30_U (e,f). Red arrow and red circles signal different anomalies in data that are mitigated by the DEM composite. AGBH values are mapped from 0 (black-blue) to 25m and more (yellow).

Among the different DEM considered in the study, AW3D30 was produced by processing the most accurate optical satellite data input (2.5 m spatial resolution) and the DEM product was available at the highest spatial resolution as well (30 m). SRTM30 and AW3D30 are the DEM sources that performed better in estimating the GVC of built-up areas, with AWD3D30 slightly outperforming SRTM30 with some exceptions due to site-specific issues. For example, in London, the AW3D30 data suffers from a blurring effect (see Fig 6C) on half of the test area. In New York, the SRTM30 shows exceptionally poor performances (see Fig 6B), likely associated to areas where backscattering radar signal suffered from shadow effects induced by the presence of high-rise buildings [3]. These shadowed areas create patches of “no data” that were filled with interpolated values during the processing of the SRTM30 DEM [94].

The two DEM composites CMP_SRTM30-AW3D30_U and CMP_SRTM30-AW3D30_I perform well across different case studies, with CMP_SRTM30-AW3D30_U at least as good as the best original DEM sources. This suggests that omission error in estimating of building heights with respect to the surrounding terrain elevation may be dominant in the global DEMs included in the study. The composite CMP_SRTM30-AW3D30_U based on point-wise maxima operator was found to be the most appropriate. Being the SRTM30 and AW3D30 DEMs derived from microwave radar vs. optical sensor technology, they incorporate different patches of interpolated DEM data. This makes the two datasets complementary, further justifying the use of the composite (see Fig 6E and 6F).

Fig 6 shows an example of the AGBH at 250 m spatial resolution estimated by univariate regression for London and New York.

Table 3 shows the five best performing GSFs, extracted from the CMP_SRTM30-AW3D30_U composite and their errors in estimating the four GVCs of built-up areas. The GSFs that best estimate the Gross Building Height (GBH) parameters exhibit a similar RMSE. Moreover, the generalization by the mean operator produces the best performing GSFs. Finally, the best GSFs for estimation the GBH are all based on second order discrete derivative made by chaining of Morphological and Laplacian first-order derivative filtering. The most-performing second-order derivative spatial filtering it is done by setting small neighbourhood of 3x3 and 5x5 DEM grid cells, thus emphasizing the high-frequency spatial information of the DEM related to the presence of built-up structures vs. the low-frequency spatial information of the DEM related to the variation of the terrain surface. Accordingly to these results, it can be observed that the most performing GSF for linear prediction of GBH are based on the *spatial generalization by the mean of the second-order discrete derivative of the DEM targeting high-frequency DEM spatial information*.

**Table 3:** The five best performing GSF extracted from the CMP_SRTM30-AW3D30_U composite and their errors expressed as RMSE by type of GVC (gross vs. net) of built-up areas.

Gross Building Height				Net Building Height			
AGBH		SGBH		ANBH		SNBH	
best DSM GSF	RMSE	best DSM GSF	RMSE	best DSM GSF	RMSE	best DSM GSF	RMSE
MEAN_f1d1	2.925	MEAN_f1d2	3.925	MEAN_ptxiw11	7.584	MEAN_f2d2	4.568
MEAN_f3d1	2.944	MEAN_m2d2	3.929	MEAN_ptxuw11	7.593	MEAN_f1d2	4.635
MEAN_f2d1	2.946	MEAN_f1d1	3.930	MEAN_m1	7.625	STD_m2	4.753
MEAN_m2d2	2.948	MEAN_f2d1	3.930	STD_f1	7.631	STD_m1	4.762
MEAN_m2d1	2.954	MEAN_m2d1	3.934	STD_m1	7.637	STD_f1	4.770

Note: AGBH = Average of Gross Building Height, SGBH = Standard Deviation of the Gross Building Height, ANBH = Average of Net Building Height, SNBH = Standard Deviation of the Net Building Height.

Concerning the Net Building Height (NBH) estimation, the best performing GSFs exhibit a more heterogeneous performance as compared to the GBH estimation. The generalization by standard deviation operator produces the best performing GSFs, and most of the best performing GSFs are calculated as first-order discrete derivative made by Morphological or Laplacian filtering in the DEM data grid domain. Also in this case, the best-performing spatial filtering it is done by setting small neighbourhood of 3x3 and 5x5 DEM grid cells, thus targeting high spatial frequency information in the DEM data. Accordingly to these results, the most performing GSF operators for linear prediction of NBH are based on the *spatial generalization by standard deviation of the first-order discrete derivative of the DEM targeting high-frequency DEM spatial information*. Table 4 shows the intercept and the slope coefficient of the univariate regression relatively to the DEM GSF “MEAN_m2d1” and “STD_m1” that are the best performing in estimating the GBH and NBH, respectively, from the CMP_SRTM30-AW3D30_U DEM composite.

**Table 4:** Intercept and slope coefficient of the univariate linear regression for estimation of the GVCs from the best two GSFs.

*GVC*	*Intercept*	*Slope*	*DEM GSF*
AGBH	0.088566	0.466495	MEAN_m2d1
SGBH	1.040022	0.709511	MEAN_m2d1
ANBH	6.136539	4.223892	STD_m1
SNBH	0.150463	3.643446	STD_m1

### 4.2 Multivariate regression analysis

#### 4.2.1 Pairwise correlation among input predictors

This part of the study analyses 67 GSF predictors; 60 GSF derived from the DEM composite CMP_SRTM30-AW3D30_U, and additional 7 GSF derived from anthropogenic land use (built-up areas) and vegetation presence (normalized vegetation index) as extracted from global collections of Landsat imagery at 30 m of spatial resolution.

**Fig 7:**
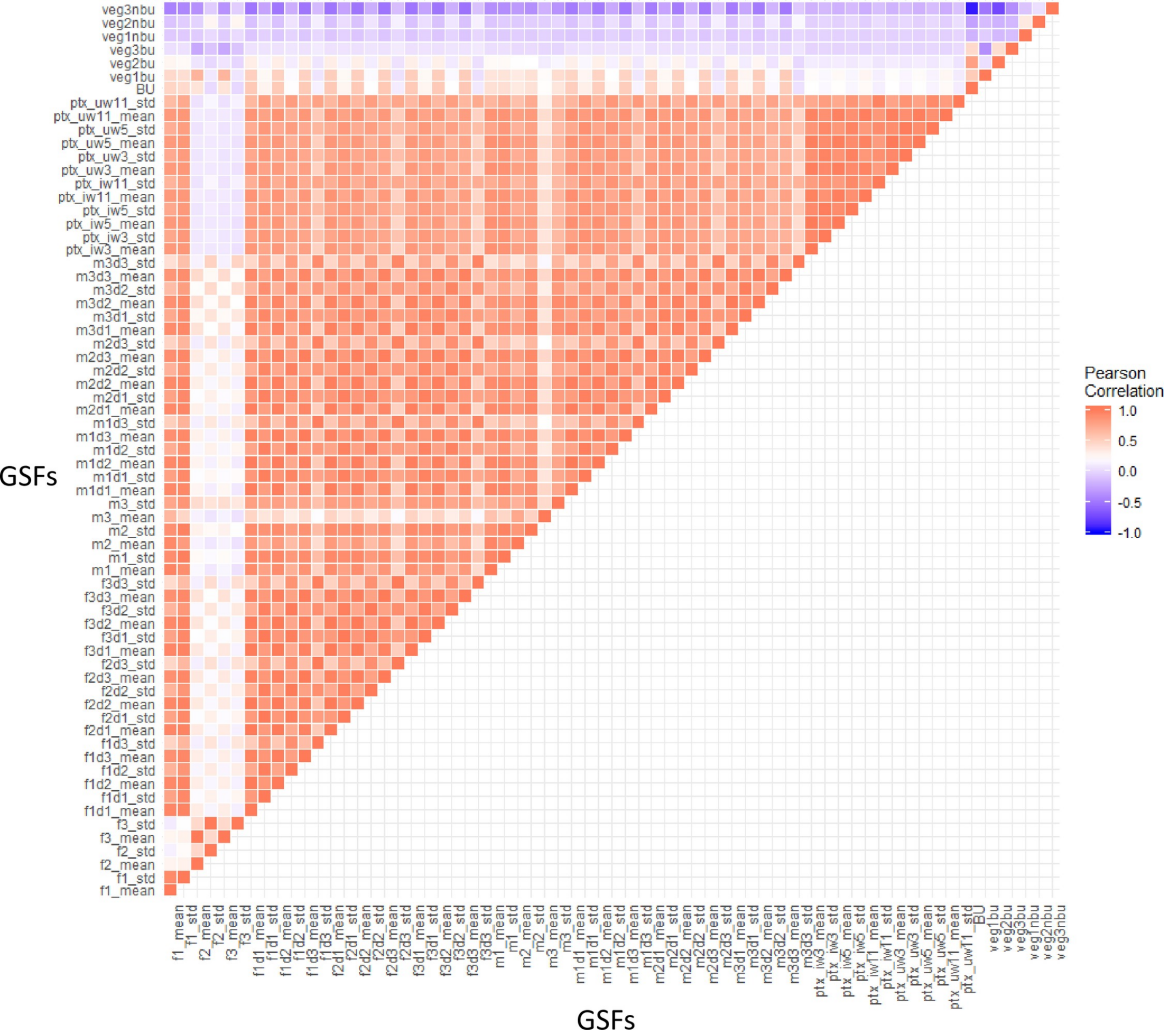
Correlogram between GSF.

The pairwise correlations between the 67 GSF predictors are shown in Fig 7. Strong positive correlations are observed between the different features derived from the DEM composite, in particular between the Laplacian convolutional filtering with different central weighting parameter when generalized with the standard deviation operator (e.g. f1_std, f2_std, and f3_std), or between their discrete local derivative calculated with morphological gradient operators in the local neighboring of 3x3 pixels also generalized with the standard deviation operator (e.g. f1d1_std, f2d1_std, and f3d1_std). The high green surfaces outside the built-up area (veg3nbu) correlate negatively with the built-up surface (BU) and with the high green surfaces inside the built-up areas (veg3bu). This was expected from the known inverse relationship between increasing built-up areas values and NDVI values [95]. To solve the multicollinearity of predictors, a stepwise dimensionality reduction process is performed. At each iteration, the mean absolute correlation *μ*|*r*| of each variable respect all the others is derived from the pairwise correlation table, and the variable with the largest *μ*|*r*| is excluded from the analysis under the condition that *μ*|*r*| is greater than a given cut-off value. For this purpose, a cut-off value of *μ*|*r*| > 0.85 was defined. As a result of this analysis, out of 67 predictors originally assessed, only 16 were considered to subsequently build the MLR.

#### 4.2.2 MLR and selection of variables

The results of the MLR are summarized in Table 5. The results confirm the findings of the univariate regression analysis, showing that the considered GVCs perform better in the prediction of GBH than for NBH. This is evidenced by a higher adj. R^2^ 0.57 and 0.63 against 0.49 and 0.55, respectively, and lower RMSE of 2.4 and 3.25 against 6.63 and 4.38, respectively, as well as a lower RSME from cross validation (RSME_CV).

**Table 5:** Intercept and coefficients of the MLR models used for estimating the GVCs considered in the study.

	*AGBH*	*ANBH*	*SGBH*	*SNBH*
(Intercept)	2.07 ***	11.05 ***	3.87 ***	4.39 ***
	(0.01)	(0.03)	(0.01)	(0.02)
f1_std	0.87 ***	4.46 ***	2.16 ***	3.56 ***
	(0.04)	(0.12)	(0.06)	(0.08)
f2_mean	-1.28 ***	-3.99 ***	-1.66 ***	-1.12 ***
	(0.01)	(0.04)	(0.02)	(0.02)
m1_mean	-0.65 ***	-2.22 ***	-0.69 ***	-0.20 **
	(0.04)	(0.11)	(0.05)	(0.07)
m2_mean	0.96 ***	2.20 ***	1.01 ***	0.41 ***
	(0.03)	(0.08)	(0.04)	(0.05)
m2_std	-0.64 ***	-0.51 ***	-0.43 ***	-0.21 **
	(0.04)	(0.11)	(0.05)	(0.07)
m3d2_mean	2.33 ***	1.70 ***	2.02 ***	0.94 ***
	(0.03)	(0.08)	(0.04)	(0.05)
m3d2_std	-0.79 ***	-0.73 ***	-0.76 ***	-0.27 ***
	(0.02)	(0.07)	(0.03)	(0.04)
ptx_uw5_std	-0.10 ***	0.02	-0.09 *	-0.23 ***
	(0.03)	(0.08)	(0.04)	(0.05)
ptx_uw11_std	0.50 ***	1.38 ***	0.88 ***	1.28 ***
	(0.02)	(0.07)	(0.03)	(0.05)
BU	-0.34	-3.33	-1.70	-2.12
	(1.22)	(2.73)	(1.65)	(1.80)
veg1bu	1.12 *	3.29 *	1.31	1.24
	(0.53)	(1.57)	(0.71)	(1.03)
veg2bu	0.46	1.54	0.93	0.94
	(0.46)	(1.19)	(0.62)	(0.78)
veg3bu	-0.06	1.59	0.19	0.49
	(0.35)	(0.99)	(0.48)	(0.65)
veg1nbu	-0.07	0.35	-0.34	-0.13
	(0.25)	(0.37)	(0.34)	(0.24)
veg2nbu	0.03	-0.59	-0.25	-0.30
	(0.19)	(0.41)	(0.25)	(0.27)
veg3nbu	-0.04	-1.44	-1.27	-0.82
	(1.06)	(2.35)	(1.44)	(1.55)
*n*	59566	59566	59566	59566
*RMSE*	2.40	6.63	3.25	4.38
*RMSE_CV*	2.65	6.64	3.55	4.37
*adj*. *R2*	0.57	0.49	0.63	0.56

Note

• GVCs include: AGBH Average of Gross Building Height, ANBH Average of Net Building Height, SGBH Standard deviation of the Gross Building Height, and SNBH Standard deviation of the Net Building Height.

• The standard error of the coefficients in parenthesis.

• The p-value notation of the statistical significance: high *** p < 0.001; medium ** p < 0.01; low * p < 0.05.

• n is the number of valid samples.

• RMSE is the root mean square error considering all the samples.

• RMSE_CV is the RMSE of cross-validation separating training set vs. testing set samples.

The fact that both the RMSE and the RMSE_CV are almost equal suggests that the four MLR models considered don’t over fit and the data given the number of explanatory variables involved vs. the number of observations that are several orders of magnitude greater (16 vs. ~60000).

The results also show that not every GSF equally contributes to the improvement of the MLR predictions. These results indicate that almost all GSFs derived from DEM have statistically significance, with the exception of the ptx_uw5_std feature in the assessment of ANBH. Inversely, almost all the ancillary GSF variables have a low statistical significance except for low green surface in the built-up area (veg1bu), which is relevant for estimating both the gross and net mean building heights (AGBH, ANBH respectively).

**Fig 8:**
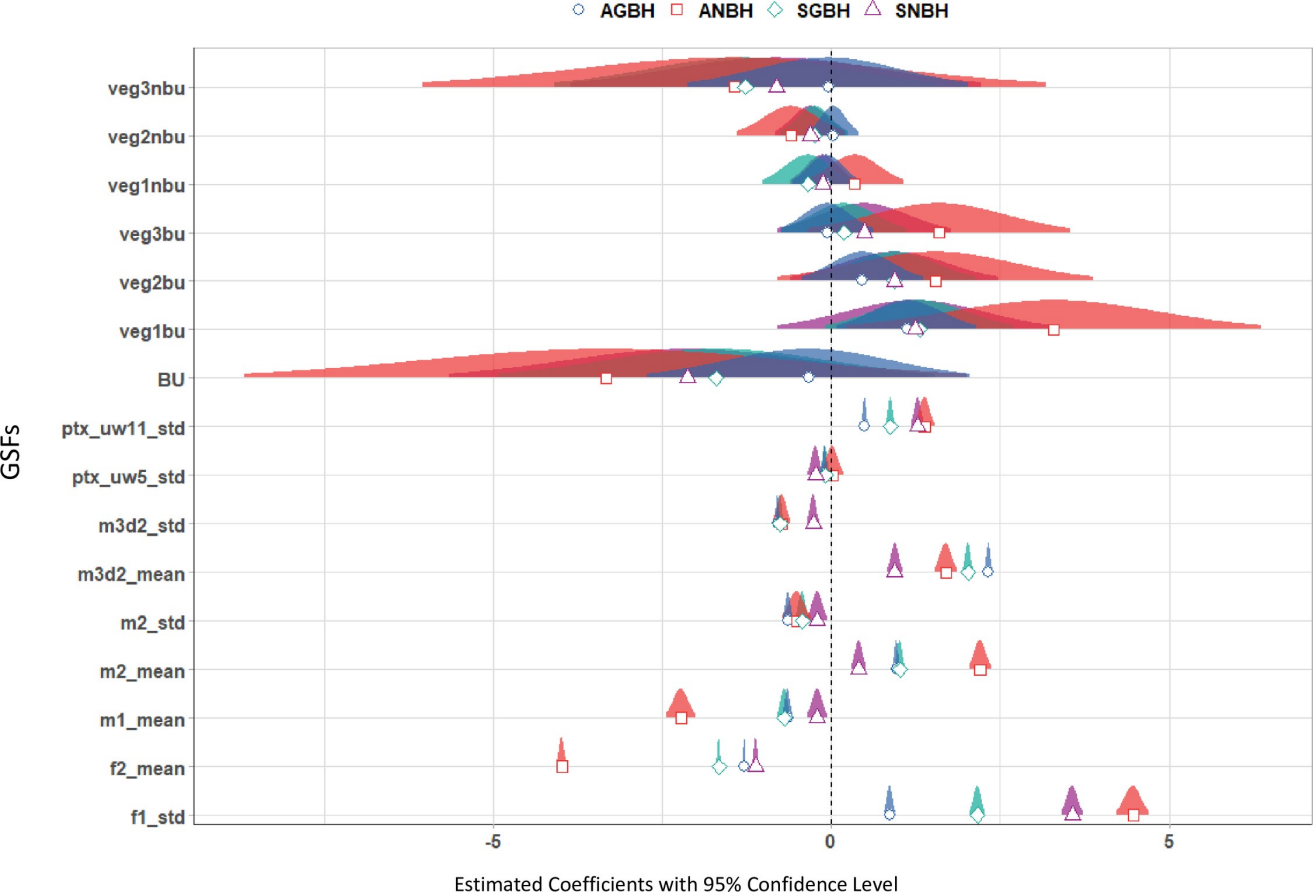
Estimated coefficients of the individual predictors and the associated uncertainties for each of the four models estimating the GVC of built-up areas.

Fig 8 shows the regression coefficients of the four models associated with each of the four GVCs of built-up areas considered in the study, represented together with their confidence intervals for a confidence level of 95%. The coefficients plotted in Fig 8 describe the higher or lower effect of each variable on the estimated GVCs of built-up areas.

From the values and the distribution of the coefficients, it could be inferred that:

- The confidence intervals of almost all the coefficients of the DEM GSFs, with the exception of ptx_uw5_std, do not include 0 which give some evidence that they are relatively more useful for estimating the GVC of built-up areas considered in the study. Inversely, most of the ancillary GSFs (i.e. vegetation and built-up densities) include 0, with the exception of veg1bu, indicating that they are generally less useful for assessing the GVCs of built-up areas.
- the DEM derived GSF f1_std has the strongest positive linear relationship with ANBH and SNBH.
- the DEM derived GSF f2_mean and m1_mean show a strong negative linear relationship with ANBH.

#### 4.2.3 Relative importance of the predictors

**Fig 9:**
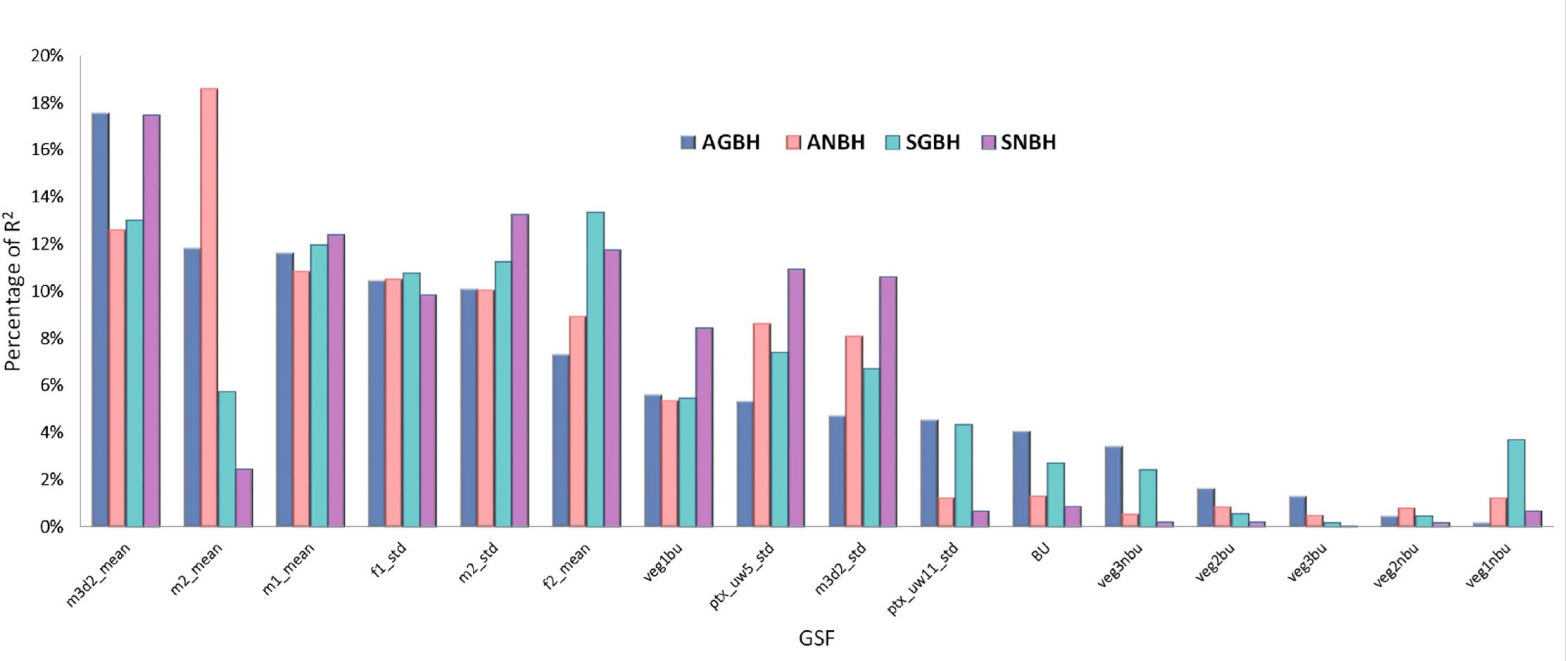
The proportion of the R^2^ contributed by each individual predictor in the four models associated with the four GVCs of built-up areas.

Based on LMG metric, Fig 9 shows that the three main GSFs that contribute the most to the assessment of the GVC of built-up areas are derived from the DEM and correspond to m3d2_mean, f2_mean and f1_std. It should be noted that the heterogeneity of these best performing GVCs captures different aspects of the DEM spatial information (Table 2). This suggests that there is no simple relationship between the net or gross building heights and the size of the neighbouring used in the spatial filtering, and that a combination of different spatial filtering of the same DEM data is required to improve the automatic detection of built-up structures. These findings are in line with other studies [96].

Regarding the ancillary GSFs (i.e. built-up densities and green surfaces), the results reveal that although the contribution of built-up densities (BU) are not statistically significant, they account for 4% of the variance in the estimation of the AGBH and 4.3% in the SGBH. The remaining GSFs associated with green surfaces together explain 12.5% of variance in the assessment of the AGBH and 10% in the assessment of the SGBH. In the case of ANBH and SNBH, the green surfaces account for 6% and 2% of the variance respectively. These results suggest that the decision to exclude these ancillary GSFs based only on their poor estimated statistical significance would have a non-negligible cost in terms of decreasing of explained data variability.

In order to further support the decision about the ancillary GSF variables to be included or not in the final model, a stepwise selection was applied. Table 6 shows the different AIC values for the initial models including all of the 16 GSF variables and the final model with the selected minimal number of GSF variables. It can be noted that the differences between the AIC of the original models including all GSF variables and the final models including a minimal subset of the GSF variables are negligible. For the AGBH, the initial model with 16 variables is robust to changes in the number of predictors (i.e. the change in AIC is equal to 0). The most parsimonious models are the ones describing the ANBH and the SNBH with 13 selected variables.

**Table 6:** Summary results of the stepwise regression for comparing different models using a sequential replacement of GSF.

	*AGBH*	*ANBH*	*SGBH*	*SNBH*
***Initial model (all 16 variables)***	131201	225526	176604	175995
***Model_1 (15 variables)***	131201	225526	176603	175995
***Model_2 (14 variables)***	131201	225526	176601	175992
***Model_3 (13 variables)***	131201	225524	176601	175991

The values in the table represent the AIC used for selecting the best subset of variables.

#### 4.2.4 Final set of variables

A linear model can over-fit the training data if the number of explanatory variables is comparable to the number of observations. In the linear model discussed here the maximum number of GSF variables is 16 vs. a number of observations in the order to 60 thousands. As a consequence, the risk of over-fit can be considered negligible even by including in the model all the 16 GSF variables passing the collinearity test. The cost of retaining only a subset of the all 16 GSF variables in terms of decreased capacity to explain the data variability is not justified by the expected negligible benefit in terms of decreased computational complexity or increased generality of the model. Accordingly, the decision to confirm all the 16 GSF variables in the final formulation of the model was taken.

## 5) Discussion

In this study, five global DEM sources available in the open scientific domain plus two DEM composite were used to estimate the GVCs of built-up areas summarized at the spatial resolution of 250 m. Convolutional, morphological, and textural filtering of the DEM data available at circa 30 m and 90 m spatial resolution are generalized at the 250 m resolution and tested as GSF variables predicting the GVC of built-up areas, in a spatial analytical modelling framework [17–19] perspective based on linear regression.

More specifically, four distinct GVCs of built-up areas are evaluated in the study as statistical parameters to be collected from the global DEM at 250 m resolution. They are the AGBH, the ANBH, the SGBH, and the SNBH.

The study shows that the best estimation of the *net* GVC of built-up areas (ANBH, SNBH), always contains a greater error than their corresponding *gross* GVC estimation (AGBH, SGBH), both in terms of the mean and the standard deviation.

This finding is independent of the specific DEM. Presumably, the net GVC underperformance may be related to implicit limitations of the DEMs in providing information at the scale of the built-up structures. Other studies showed that built-up areas are statistically dominated by roofed surfaces at the scale of 10 m [61]. Having a grid cell size larger than the average horizontal size of roofed built-up structures as detected from EO data, the global DEMs give a better estimation of GBH than the NBH, since the NBH formulation it is based on the precise delineation of the statistical support domain (∀ *x_i_*∈*N*|*x_i_*>0) at greater resolution than the 250 m, making the NBH relatively more sensitive to the scale than the GBH.

Regarding the DEM-derived features using convolutional, morphological and textural filtering at various neighbouring sizes, the study shows that no simple relationship can be established between the DEM GSF characteristics and the GVCs of built-up areas. Nevertheless, the best 5 single predictors of the gross GVC of built-up areas are all derived by first-order statistic (mean generalization operator) of second-order derivative of the DEM data using morphological and convolutional (Laplacian) approach applied to neighbouring of 3x3 and 5x5 DEM pixels. The best 5 single DEM GSF predictors of net GVC of built-up areas are relatively more heterogeneous: they include second-order statistic (standard deviation generalization operator) of first-order derivative of the DEM data using morphological and convolutional (Laplacian) approach applied to neighbouring of 3x3 DEM pixels, but also first-order statistic (mean generalization operator) of textural contrast measurements done using a window size of 11x11 DEM pixels.

Regarding the suitability of the global DEMs for estimating the GVC of built-up areas at 250 m resolution, the study shows that the most performing DEMs are the SRTM30 and the AW3D30. Their composite CMP_SRTM30-AW3D30_U based on the point-wise maxima of SRTM30 and AW3D30 DEM-derived GSF is the most performing dataset regarding the error minimization in the six cities considered in the study.

By adopting a univariate linear regression approach on CMP_SRTM30-AW3D30_U input, the best DEM-derived Generalized Satellite Feature for estimation of the gross GVC from this DEM composite is the “MEAN_m2d1” that is the morphological gradient (dilation minus erosion with a structuring element 3x3), of the open and closing residual (with a structuring element 5x5), generalized at 250 m resolution by the mean statistical parameter. In the same univariate approach, the best DEM-derived Generalized Satellite Feature for estimation of the net GVC is the “STD_m1” that is the morphological open and closing residual (with a structuring element of 3x3), generalized at 250 m resolution by the standard deviation statistical parameter. They would estimate AGBH and ANBH with a RMSE of 2.92m and 7.58m, respectively. The same would estimate SGBH and SNBH with a RMSE of 3.92m and 4.56m, respectively.

**Fig 10:**
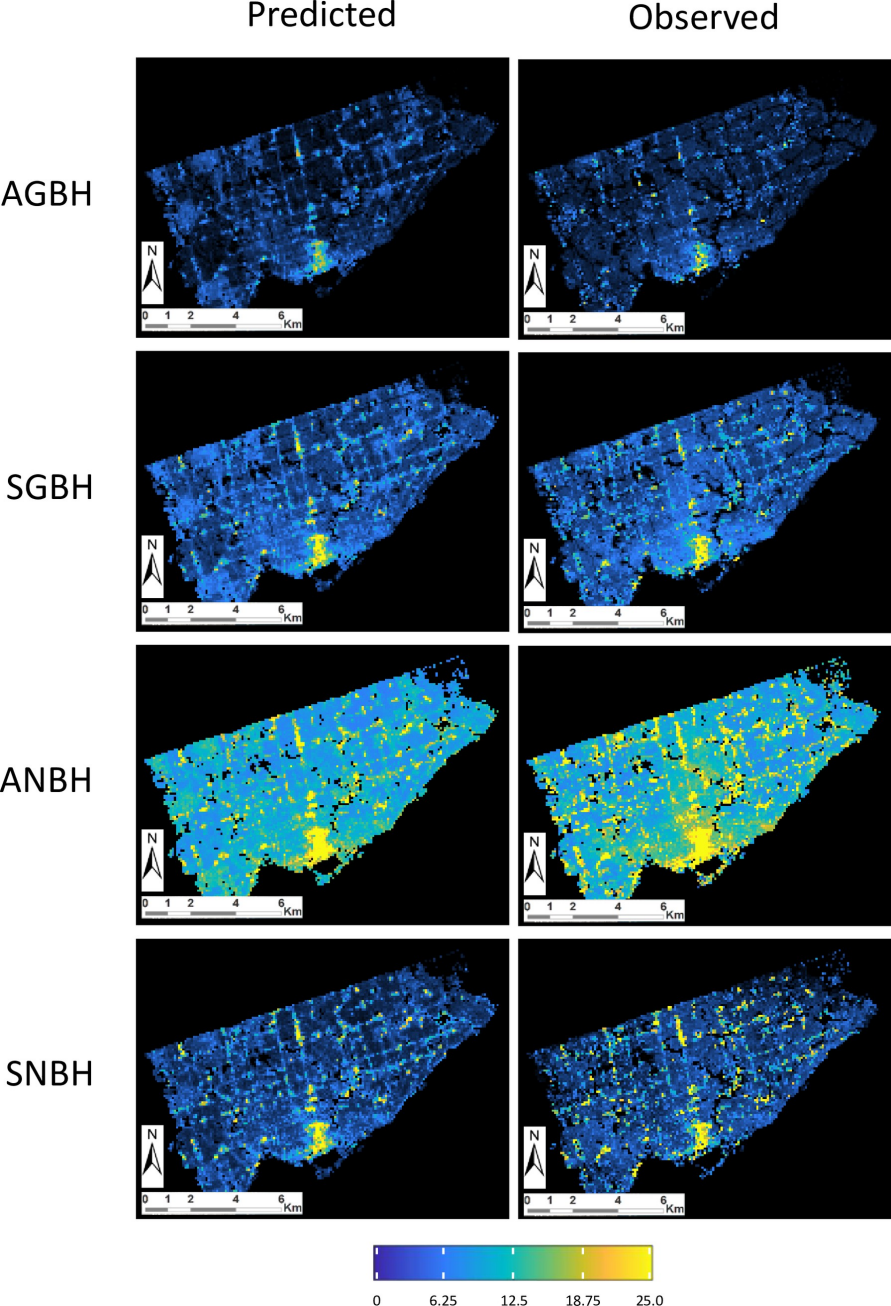
**Example of predicted (left) vs. observed (right) GVCs values at 250 m resolution using the MLR on the 16 GSFs selected in the study, as resulting in the test area of Toronto (33 X 44 km).** From top to bottom row: AGBH, SGBH ANBH, and SNBH. GVC values are mapped in the colour range from 0 (black-blue) to 25m and more (yellow).

By adopting a multivariate linear regression approach, 16 GSFs were selected for both gross and net GVC estimates, from a total list of 67 GSFs evaluated in the study as independent variables. The 16 selected GSFs include DEM-derived GSFs and ancillary GSFs aggregated from Landsat-satellite-derived information reporting about the presence of built-up areas and vegetated surfaces that are available at 30 m of spatial resolution. In this approach, an overall RMSE of 2.40m and 6.63m was assessed for AGBH and ANBH, respectively. Similarly, an overall RMSE of 3.25m and 4.38m was assessed for the SGBH and SNBH, respectively. The overall RMSE reduction resulted by the application of the multivariate vs. the univariate linear framework resulted in -0.52m and -0.95m for AGBH and ANBH, respectively. Similarly, by the adoption of the multivariate approach an overall RMSE reduction of -0.67m and -0.18m was assessed for the SGBH and SNBH, respectively. These results show that global DEM sources may be used to derive statistically generalized parameters describing the vertical characteristics of built-up areas, at the 250 m spatial resolution. Those parameters need to be tested under the requirements of specific target applications. Fig 10 compares observed vs. predicted GVCs of built-up areas by the application of the MLR on the 16 GSF selected in the study, as resulting in the test area of Toronto.

**Fig 11:**
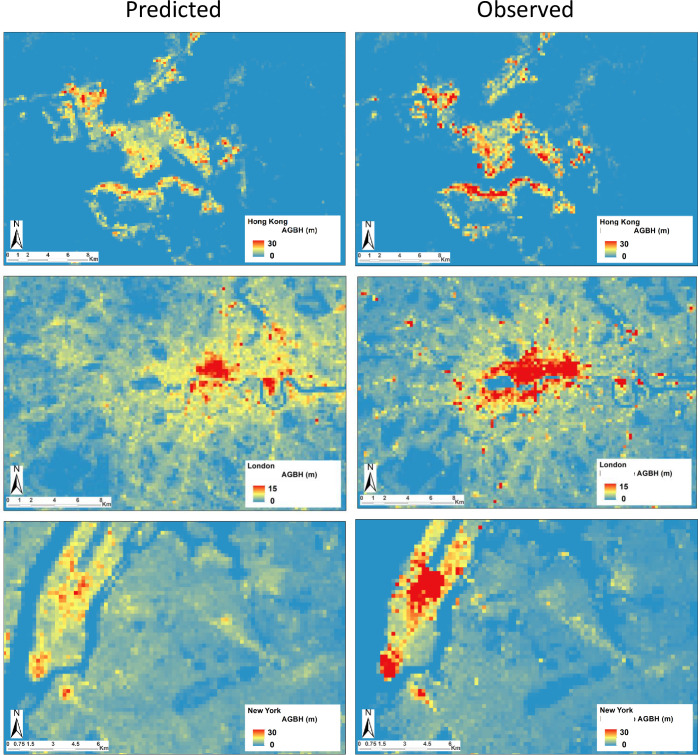
**Example of predicted (left) v. observed (right) values of AGBH by using the model proposed in the study.** From top to bottom, data extracted from the test case of Hong Kong, London, and New York.

**Fig 12:**
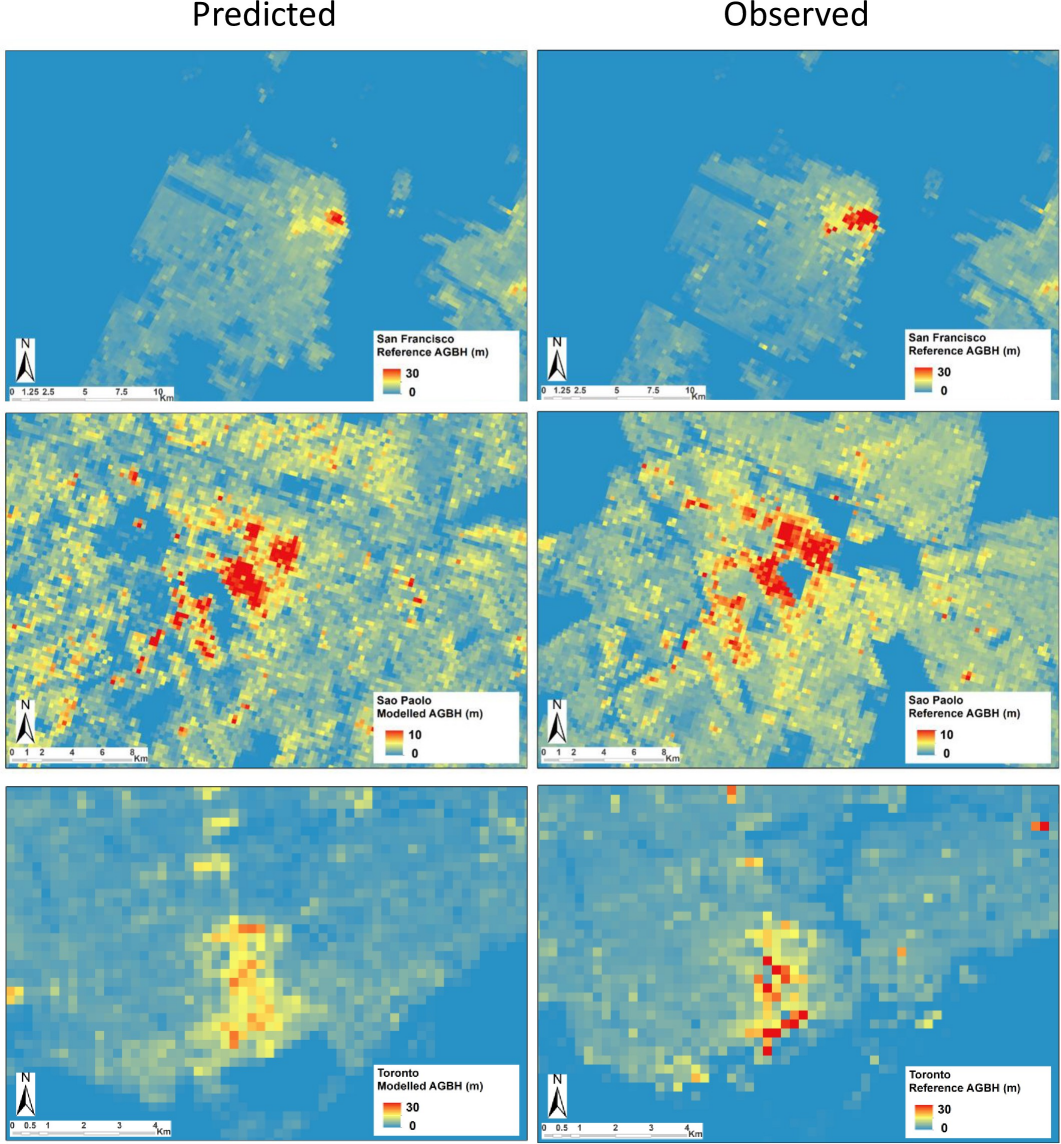
**Example of predicted (left) v. observed (right) values of AGBH by using the model proposed in the study.** From top to bottom, data extracted from the test case of San Francisco, Sao Paolo, and Toronto.

Figs 11 and 12 show and example of predicted vs. observed AGBH values in the six test cases considered in the study by application of the proposed multivariate linear model. As can be observed in the images, the hierarchy of low vs. high urban density patterns measured by the AGBH in the single city it is respected. Moreover, anomalous high levels of the predicted AGBH are not noticeable, suggesting that the model is producing a conservative prediction of the GVC of built-up areas. This prudent behaviour of the model is presumably inherited by the combination of two factors including i) the dominant omission error in the input DEM data and ii) the applied linear regression function. A conservative model prediction may be a positive feature for some applications requiring robust estimation in global data scenarios connoted by potentially high error rates or local anomalies. Nevertheless, it can be noticed that the model being calibrated in the whole set of considered data, has clear limitation in predicting exceptionally high values of the AGBH. These limitations are particularly perceptible in the Hong Kong and New York test cases, connoted by the highest density of very high-raise buildings considered in the study.

## 6) Conclusions

Five global DEMs derived from spaceborne sensors and available in the frame of the Global Earth Observation System of Systems (GEOSS) [3] are tested for their capacity to predict four GVCs of built-up areas (AGBH, ANBH, SGBH, and SNBH) at a spatial resolution of 250x250 meters, under a general linear regression analytical framework. This choice it is justified by the need of computational simplicity and the need for generality and applicability of the model in global data scenarios.

The study was conducted on six different cities where detailed reference data on the height of the buildings was available: namely Hong Kong, London, New York, San Francisco, Sao Paulo, and Toronto. All these cities are connoted by a strong presence of high-rise buildings and dense urban environment. In the frame of the study, 60 DEM-derived GSFs are tested. They correspond to 30 different spatial filtering of the DEM data and 2 statistical parameters (mean, standard deviation) tested for generalizing the DEM-derived high-spatial-frequency information at the 250 m spatial resolution.

The composite CMP_SRTM30-AW3D30_U collecting the point-wise maxima of SRTM30 and AW3D30 DEM-derived GSF was identified as the most performing solution regarding the error minimization across the six cities considered in the study. By adopting a univariate linear regression based on the best GSF derived from this DEM composite would estimate AGBH and ANBH with a RMSE of 2.92m and 7.58m, respectively. The same would estimate SGBH and SNBH with a RMSE of 3.92m and 4.56m, respectively.

By adopting a multivariate linear regression approach, 16 GSFs including DEM-derived GSFs and ancillary GSFs characterizing the presence of built-up surfaces and vegetation, would result in an improved overall RMSE of 2.40m and 6.63m for AGBH and ANBH, respectively. Using the same multivariate approach, an overall improved RMSE of 3.25m and 4.38m was assessed for the SGBH and SNBH, respectively.

More advanced DEM data filtering techniques may help in the limitations found in the study and should be investigated. Moreover, other multivariate regression frameworks may have the potential to improve the models discussed here exclusively in the frame of the linear least square regression, and could be investigated in the future. Furthermore, the introduction of better spatial resolution ancillary GSF data in the multivariate regression should be tested. For instance Sentinel satellite sensors could be used to generate data composites characterizing vegetation presence from calibrated reflectance data [97] or characterizing the presence of built-up areas [98–100]. The delineation of built-up areas from both Sentinel 1 radar data [99] and Sentinel 2 optical data [98, 100] has a higher spatial resolution as compared to the Landsat data tested in this study. Consequently, an improvement would be expected especially regarding the estimation of net vertical components of built-up areas, which are more dependent on the spatial resolution than the gross vertical components.

The DEM used in this study were acquired in one point in time and can only be used to quantifying built-up volumes in the same point in time. The global and spatially-explicit data estimating the built-up volume for one point in time is not existing today with a spatial resolution of 250 m as discussed in this study. This gap could be filled by the application of the models discussed here to global baseline data available in the open and free scientific domain. The approach discussed in this study, however, is not suitable for monitoring changes in the vertical components of cities as required in international policy frameworks. High-spatial-frequency volumetric information which is characterized by relatively higher changes rates, would be useful in the context of monitoring changes. In this perspective, the improvement of the frequency of update of global DEMs would be necessary, especially focusing on the areas where urbanization processes are more intense and/or the volumetric characteristics of the urban environment are changing quickly. This study contributes to future efforts in monitoring changes in building volume and for improving the understanding of the continuous urbanization processes at global scale for achieving sustainable development.

## Supporting information

S1 File (DOCX)
