# Supplementary material for: Generalized Vertical Components of built-up areas from global Digital Elevation Models by multi-scale linear regression modelling
Source: PLoS One. 2021 Feb 10;16(2):e0244478. doi: 10.1371/journal.pone.0244478 (PMC7875370; doi:10.1371/journal.pone.0244478)
Supplement: S1 File — (DOCX) [file pone.0244478.s001.docx]

**PONE-D-19-25546**

**S1 File**

Contents

[Technical details on the DEM data used in the study 1](#_Toc57189641)

[Mathematical details on the calculation of the DEM-derived GSF 3](#_Toc57189642)

[Discrete convolutional filters (f1 f2 f3) 3](#_Toc57189643)

[Morphological Filters 3](#_Toc57189644)

[Morphological gradient (d1, d2, d3) 3](#_Toc57189645)

[Composite of Opening and Closing residuals (m1, m2, m3) 3](#_Toc57189646)

[Textural Measures (ptx i/u w3/w5/w11) 4](#_Toc57189647)

[General list of DEM spatial filters 6](#_Toc57189648)

[Mathematical details on the Generalized Vertical Components of the built-up areas 7](#_Toc57189649)

[Definition of the Generalized Vertical Components of built-up areas 7](#_Toc57189650)

[Algebra of the built volume 8](#_Toc57189651)

[Correlation between DEM-derived GSFs and the GVC of built-up areas 10](#_Toc57189652)

[References 14](#_Toc57189653)

# Technical details on the DEM data used in the study

**“SRTM30” and “SRTM90”.** The Shuttle Radar Topography Mission (SRTM) at 1 and 3 arcseconds: the data used for producing the SRTM was collected from two interferometers C-band and X-band systems at 1-arcsecond (~30 meters at the equator) [1] aboard the Space Shuttle Endeavour launched in 2000. It covers land surfaces between 60 degrees north latitude and 56degrees south latitude. The 3-arcsecond DEM (~90m at the equator) was created by 3 x 3 averaging of the 1-arc second data. At a global scale, the 1-arcsecond version (STRM90) has the same root-mean-square error (RMSE) of 10.3 m as its 3-arcsecond version (SRTM30) [2] ranging from 5.9 m in urban areas to 10.4 m in bushland [3]. It is available from NASA’s Earth Explorer website [4].

**“ASTER”.** The Advanced Spaceborne Thermal Emission and Reflection Radiometer (ASTER) Global Digital Elevation Model (ASTER_GDEM): it was developed jointly by Japan’s Ministry of Economy, Trade and Industry (METI) and the United States National Aeronautics and Space Administration (NASA) from ASTER stereo data acquired between 2000 and 2008. The ASTER instrument has an along-track stereoscopic capability using its near infrared spectral band and its nadir- and backward-viewing telescopes to acquire stereo image data with a base-to-height ratio of 0.6. The ground sampling distance is 15 meters (m) in the horizontal plane [5]. ASTER_GDEM product covers land surfaces between 83 degrees north latitude and 83 degrees south latitude. The vertical accuracy changes with location [6] and is influenced by the land cover type. It is estimated to be around ±7.97 m for version 2 [7] in built-up areas.

**“AW3D30”**. The Advanced Land Observing Satellite (ALOS) World 3D–30 m (AW3D30): It was photogrammetrically derived from visible-band, 2.5-m resolution stereo images acquired by the Panchromatic Remote-sensing Instrument for Stereo Mapping (PRISM) on board the ALOS spacecraft from 2006 to 2011. This DEM has the resolution of 1 arc-second and therefore is commonly considered an alternative to SRTM and ASTER_GDEM. One major shortcoming of AW3D30, is related to data gaps as a result of cloud cover and gaps between satellite orbits [8]. Preliminary validation results provided an RMSE of 4.40 m for height accuracy [8].

**“MERIT”**. The Multi-Error-Removed Improved-Terrain Digital Elevation Model (MERIT_DEM): it was developed by removing multiple error components (absolute bias, stripe noise, speckle noise, and tree height bias) from the existing spaceborne DEMs (SRTM3 v2.1 and AW3D30 v1) [9]. It represents the terrain elevations at 3 arcseconds and covers land areas between 90 degrees north and 60 degrees south. The data is freely available for research and education purpose.

**“CMP_SRTM30-AW3D30_U”**. Composite of the SRTM30and AW3D30 derived features, using a point-wise maxima operator applied before the generalization step.

**“CMP_SRTM30-AW3D30_I”**. Composite of the SRTM30and AW3D30 derived features, using a point-wise minima operator applied before the generalization step.

The characteristic of the five global elevation datasets used in input of this study are summarized in Table 1.

Table 1. Characteristics of the global elevation datasets used in this study

| Name | Acquisition year | Coverage | Sensor/Satellite | | Resolution | Vertical accuracy |
| --- | --- | --- | --- | --- | --- | --- |
| SRTM30 | 2000 | 56° S- 60 ° N | | SAR C-band, 1as | 30 m | 6 m to 10.4 (RMSE) |
| SRTM90 | 2000 | 56° S- 60 ° N | | SAR C-band, 1as | 90 m | 6 m to 10.4 (RMSE) |
| ASTER | 2000-2008 | 83° S- 83 ° N | | ASTER Optical 15m | 30 m | 8 m (95% conf.) |
| AW3D30 | 2006-2011 | 82° S- 82 ° N | | ALOS Optical 2.5m | 30 m | 4.4 (RMSE) |
| MERIT | 2000-2011 | Entire Earth | | AW3D30 & SRTM | 90 m | 5m (LE90) |

# Mathematical details on the calculation of the DEM-derived GSF

## Discrete convolutional filters (f1 f2 f3)

Be O the output raster data obtained by convolving the X input data with the K convolution kernel. The raster convolution transform can be written as

$O_{(i,j)}= \sum_{k=1}^{m} \sum_{l=1}^{n} X_{\left( i+k-1,j+l-1 \right)}\cdot K_{\left( k,l \right)}$

With m,n the number of rows and columns of the kernel, (i,j) the row,column position index in the input and output raster data, and (k,l) the row, column position index in the raster convolution kernel.

In the study, three options for the K convolutional kernel were tested, assuming a 3x3 Laplacian kernel

$\begin{matrix} -1 & -1 & -1 \\ -1 & w & -1 \\ -1 & -1 & -1 \end{matrix}$ With *w* = 8,9,10 for f1, f2, f3, respectively, as noted in Table 2.

## Morphological Filters

The Morphological filters applied in the study belong to the grey-level mathematic morphology by using flat structuring elements that are symmetric respect to the central point as introduced in [10]

### Morphological gradient (d1, d2, d3)

The morphological gradient of the function$f$ it is made by the algebraic composition of the dilation and the erosion of $f$ , and can be considered as a first discrete derivative of the function $f$ evaluating the slope of the function in a given discrete neighbourhood set by the structuring element.

The grayscale erosion of a raster spatial function $f$ involves assigning to each pixel, the minimum value found over the neighbourhood of the structuring element SE

$$\varepsilon_{SE}\left( f \right)_{x}= \left( f⊖SE \right)_{x}= \min_{\beta\in SE} f\left( x+\beta\right)$$

The grayscale dilation of a raster spatial function $f$ involves assigning to each pixel, the maximum value found over the neighbourhood of the structuring element SE

$$\delta_{SE}\left( f \right)_{x}= \left( f\oplus SE \right)_{x}= \max_{\beta\in SE} f\left( x+\beta\right)$$

The morphological gradient of $f$ it is given by the difference between the dilation and the erosion of the same input $f$ by assuming a given same structuring element SE.

$$\rho_{SE}= \delta_{SE}- \varepsilon_{SE}$$

In the study, d1, d2, and d3, denote the morphological gradient calculated by assuming a square structuring element of 3x3, 5x5, and 11x11 pixels, respectively, as noted in the Table 2.

### Composite of Opening and Closing residuals (m1, m2, m3)

The grayscale opening of a spatial function $f$ involves performing a grayscale erosion, followed by grayscale dilation. The opened value of a pixel is the maximum of the minimum value of the raster function $f$ in the neighbourhood defined by the SE:

$$\gamma_{SE}= \delta_{SE}\left( \varepsilon_{SE} \right)$$

By duality, the grayscale closing of a raster function $f$ involves performing a grayscale dilation, followed by grayscale erosion.

$$\phi_{SE}= \varepsilon_{SE}\left( \delta_{SE} \right)$$

The white top-hat (WTH) or top-hat by opening of a raster function $f$ is the difference between the original function $f$ and its opening $\gamma$

$${WTH}_{SE}\left( f \right)=f- \gamma_{SE}\left( f \right)$$

The black top-hat (WTH) or top-hat by closing of a raster function $f$ is the difference between the closing $\phi$ and the original function $f$

$${BTH}_{SE}\left( f \right)=\phi_{SE}\left( f \right)-f$$

Considering the DEM data as the input raster function $f$, the ${WTH}_{SE}\left( f \right)$ will isolate the DEM peaks or crest lines (convex parts of the DEM function) that are thinner than the SE size. By duality, the ${BTH}_{SE}\left( f \right)$will isolate the DEM sinks or valleys (concave parts of the DEM function) that are thinner than the SE size [11].

The composite of the opening and closing residuals used in the study it is constructed in order to enhance the contrast between the convex vs. concave parts of the local DEM function, at the given scale defined by the size the SE used in the transform, and it is defined as

$$m_{SE}=k+ {WTH}_{SE}\left( f \right)- {BTH}_{SE}\left( f \right)$$

With k as a constant, and the structuring element SE of 3x3, 5x5, and 11x11 pixels, for m1, m2, and m3, correspondingly, as noted in the Table 2. In the study, the $m_{SE}$ was numerically evaluated in 256 integer levels (uint8) with k=128.

## Textural Measures (ptx i/u w3/w5/w11)

The textural measures used in this study are based on the grey-level co-occurrence matrix (GLCM) as introduced in [12].

A GLCM is a matrix where the number of rows and columns is equal to the number of grey levels, *N_g_*, in the raster function $f$. The matrix element *P (i, j | ∆x, ∆y)* is the relative frequency with which two pixels, separated by a pixel distance *(∆x, ∆y)* called *displacement vector*, occur within a given neighbourhood (*window size*), one with intensity level ‘i’ and the other with intensity level ‘j’.

The matrix element P (i, j | d, ө) contains the second order statistical probability values for changes between grey levels ‘i’ and ‘j’ at a particular displacement vector defined by a distance *d* and at a particular angle (ө).

In the study, the textural filtering it is applied after the high-spatial-frequency filtering generated by the composite of the opening and closing residuals (m1, m2, and m3 transforms). Consequently, the GLCM encoded in this study are set with a number of levels *N_g_* = 256 and eight displacement vectors corresponding to the height directions in the square raster grid, with the *∆x, ∆y* equal to a combination of 0, 1, or -1 pixel as illustrated below:

$$\left( \Delta x, \Delta y \right)= \left[ \begin{matrix} \left( -1,-1 \right) & \left( 0,-1 \right) & \left( 1,-1 \right) \\ \left( -1,0 \right) & \circ& \left( 1,0 \right) \\ \left( -1,1 \right) & \left( 0,1 \right) & \left( 1,1 \right) \end{matrix} \right]$$

In the study, the Contrast (also called Moment 2 or standard deviation) textural measure was calculated based on the GLCM. The Contrast is the measure of intensity or grey level variations between the reference pixel and its neighbour set by the displacement vector. Large contrast reflects large intensity differences in GLCM and it is defined as:

$$Contrast = \sum_{i} \sum_{j} \left( i-j \right)^{2}{\cdot p}_{d}\left( i,j \right)$$

Assuming *N_g_* the number of grey levels, *p_d_* is the normalized symmetric GLCM of dimension *N_g_* X *N_g_* with a given displacement vector *d*, and the *p_d_(i,j)* is the (i,j)*th* element of the normalized GLCM.

Eight anisotropic contrast measures are obtained for each point of the filtered DEM, corresponding to the eight distinct displacement vectors considered. Two rotation-invariant composite of the anisotropic contrast measures are considered in the study based on point-wise min, max extrema of the output textural function [13]. They are noted in the study as ptxi, ptxu, correspondingly. By noting as *C_d_* the contrast textural measure obtained for a given displacement vector *d*, they are formulated as:

$$ptxi=\min_{d\in(1..8)} C_{d}$$

$$ptxu=\max_{d\in(1..8)} C_{d}$$

Moreover, three different window sizes have been tested in the textural measurements: they are 3x3, 5x5 and 11x11 pixels and are noted as w3, w5, and w11, respectively in the feature list of Table 2.

## General list of DEM spatial filters

Table 2 - The list of features derived from the DEM data. All spatial filters are computed in the DEM native grid geometry (spatial resolution, global projection, grid origin). Subsequently, they are generalized to the 250m grid cell using two statistical operators (mean, standard deviation). Consequently, 60 totals GSF are evaluated in the study.

# Mathematical details on the Generalized Vertical Components of the built-up areas

## Definition of the Generalized Vertical Components of built-up areas

The *Generalized Vertical Components* (GVC) of built-up areas aim to summarize some relevant geometric characteristics of the three-dimensional built-up environment. They statistically describe the height of the built-up surfaces assessed at a fine scale to a given, broader generalization scale. The description is done by first and second order statistics reporting about the central tendency and the variability of the built height information in the given neighboring determined by the generalization scale.

Be $x_{i}$ the measure of the building height in each point of a raster spatial data grid at the spatial resolution of 1x1 meter also called reference Digital Building Height Model, and be N a spatial generalization neighboring of size 250x250 meters. We define four GVC of built-up areas aggregating the information of *x* to the larger neighboring *N*; they are the Average Gross Building Height (AGBH), the Average Net Building Height (ANBH), the Standard Deviation of Gross Building Height (SGBH), and the Standard Deviation of Net Building Height (SNBH).

Equation 1 : Average Gross Building Height

${AGBH}^{\underset{\to}{x}N}= \left\{ \frac{1}{n}\sum_{i=1}^{n} x_{i}\forall x_{i}\in N \right\}$

Equation 2 : Average Net Building Height

${ANBH}^{\underset{\to}{x}N}=\left\{ \frac{1}{n}\sum_{i=1}^{n} x_{i}\forall x_{i}\in N |x_{i}>0 \right\}$

Equation 3 : Standard Deviation of Gross Building Height

${SGBH}^{\underset{\to}{x}N}= \left\{ \sqrt{\frac{1}{n-1} \sum_{i=1}^{n} \left( x_{i}- \bar{x} \right)^{2}} \forall x_{i}\in N \right\}$

Equation 4 : Standard Deviation of Net Building Height

${SNBH}^{\underset{\to}{x}N}= \left\{ \sqrt{\frac{1}{n-1} \sum_{i=1}^{n} \left( x_{i}- \bar{x} \right)^{2}} \forall x_{i}\in N | x_{i}>0 \right\}$

With $\underset{\to}{x}N$ denoting the spatial statistical generalization of the detailed-scale variable *x* to the broader-scale neighboring *N*. The spatial domain setting the information $x_{i}$ summarized at the generalization scale of the neighboring *N* it is the entire neighboring surface domain ($\forall x_{i}\in N)$or the part of the neighboring with an information support greater than zero ($\forall x_{i}\in N | x_{i}>0$ ). In the approach discussed here, they are called “*gross generalization*” and “*net generalization*”, respectively, and mathematically they are described as a conditional statistical generalization operator.

Figure 1 – example of the Generalized Vertical Components of built-up areas in a neighbouring N of 10x10 building height samples

## Algebra of the built volume

Be $\sigma$ the surface of the sample $x_{i}$ in the raster Digital Building Height Model, the total built up volume in the neighboring N it is defined as the integral of the built height surface model, discretized in the raster grid of reference

Equation 5 : the total built up volume m^3^ in the spatial unit N

$V^{\underset{\to}{x}N}= \left\{ \sum_{i=1}^{n} {\sigma\cdot x}_{i}\forall x_{i}\in N \right\}$

Be $\sigma_{Unit}$ the surface of the spatial unit or neighboring *N* defined as the product of $\sigma$ by the cardinality of *N*

Be $\sigma_{BU}$ the surface of the support of the Digital Building Height Model *x* where *x>0*, also called *built-up surface* in the spatial domain *N*

Thus, assuming $\sigma$ of unitary surface unit (example 1x1 m) Equation 1 can be rewritten as the ratio of total built-up volume over the spatial unit surface *m^3^/m^2^*, also called *urban density* in the spatial planning regulatory practices, yielding

Equation 6 simplified Average Gross Building Height or gross Urban Density m^3^/m^2^

$${AGBH}^{\underset{\to}{x}N}= {V^{\underset{\to}{x}N}}/{\sigma_{Unit}}$$

Similarly, Equation 2 can be rewritten as

Equation 7 simplified Average Net Building Height or net Urban Density m^3^/m^2^

$${ANBH}^{\underset{\to}{x}N}= {V^{\underset{\to}{x}N}}/{\sigma_{BU}}$$

From Equation 6 and Equation 7 we derive

Equation 8 Basic gross and net relation

$\left\{ \begin{aligned} {AGBH}^{\underset{\to}{x}N}\cdot\sigma_{Unit}=V^{\underset{\to}{x}N} \\ {ANBH}^{\underset{\to}{x}N}\cdot\sigma_{BU}=V^{\underset{\to}{x}N} \end{aligned} \underset{\to}{yields} \right.{AGBH}^{\underset{\to}{x}N}\cdot\sigma_{Unit} = {ANBH}^{\underset{\to}{x}N}\cdot\sigma_{BU}$

The above relation can be used to measure the consistency of the statistical inferential model estimating independently the ${AGBH}^{\underset{\to}{x}N}$ and the ${ANBH}^{\underset{\to}{x}N}$ values on the same spatial samples.

Moreover, being ${AGBH}^{\underset{\to}{x}N}$ and ${ANBH}^{\underset{\to}{x}N}$ estimated in a given neighboring *N* of known constant surface $\sigma_{Unit}$ , the unknown *total built-up surface* estimation $\sigma_{BU}$ m^2^ in the same neighboring *N* can be derived from the ratio of gross and net Generalized Vertical Components of built-up areas as

Equation 9 Estimation of the net built-up surface from the gross and net Generalized Vertical Components

$$\sigma_{BU}= \frac{{AGBH}^{\underset{\to}{x}N}}{{ANBH}^{\underset{\to}{x}N}} \cdot\sigma_{Unit}$$

Therefore, the estimated net *built-up surface share* in the spatial unit *N* can be calculated as

Equation 10 Estimated built-up surface share from the gross and net Generalized Vertical Components

$$\frac{\sigma_{BU}}{\sigma_{Unit}}= \frac{{AGBH}^{\underset{\to}{x}N}}{{ANBH}^{\underset{\to}{x}N}}$$

Furthermore, assuming a uniform ceiling height$h$of the housing units in the built-up structures summarized at the given spatial unit *N*, the total *floor surface* $\sigma_{Floor}$ *m^2^* in the same spatial unit *N* can be estimated as

Equation 11 Basic floor surface relation

$$\sigma_{Floor}= {V^{\underset{\to}{x}N}}/h$$

That from Equation 6 and Equation 7 can be rewritten as

Equation 12 Estimation of the floor surface from the Average Gross Building Height

$$\sigma_{Floor}= \frac{{AGBH}^{\underset{\to}{x}N}\cdot\sigma_{Unit}}{h}$$

Equation 13 Estimation of the floor surface from the Average Net Building Height

$$\sigma_{Floor}= \frac{{ANBH}^{\underset{\to}{x}N}\cdot\sigma_{BU}}{h}$$

# Correlation between DEM-derived GSFs and the GVC of built-up areas

The charts included in this section show the correlation between the GSF derived from spatial filtering of DEM data and the GVC of built-up areas at 250 m spatial resolution. The correlation is assessed by the Pearson’s Correlation Coefficient as resulting by considering in the univariate regression analysis the whole set of valid samples collected in the six tests cases. For each DEM source, in the vertical axis the correlation measure and in the horizontal axis the identifier of the GSF. The ID of the GSF are consistent with the order of the DEM filter identifier included in the Table 2 : the GSF ID in the range [1..30] are calculated by using the average generalization operator from the DEM filter ID [1..30], while the GSF ID in the range [31..60] are calculated by using the standard deviation generalization operator from the DEM filter ID [1..30], correspondingly.

DEM: SRTM90


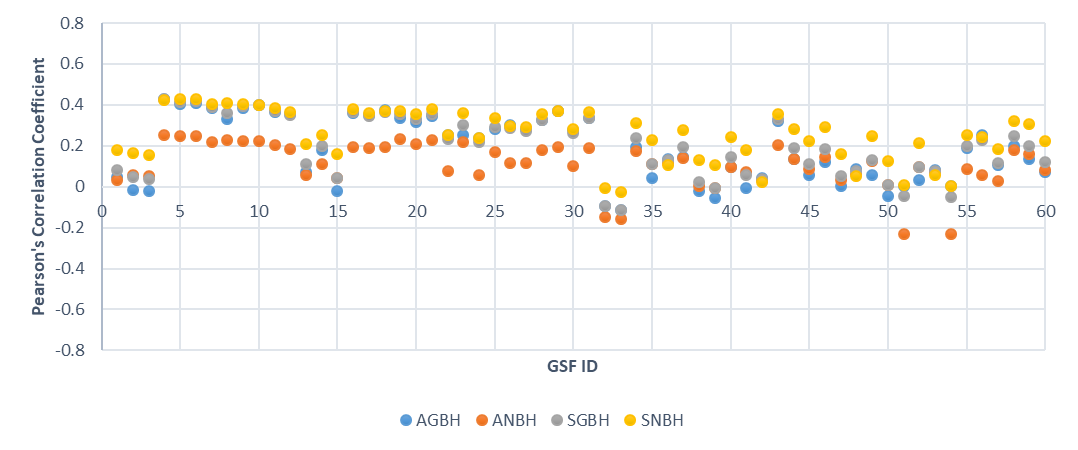


DEM: SRTM30


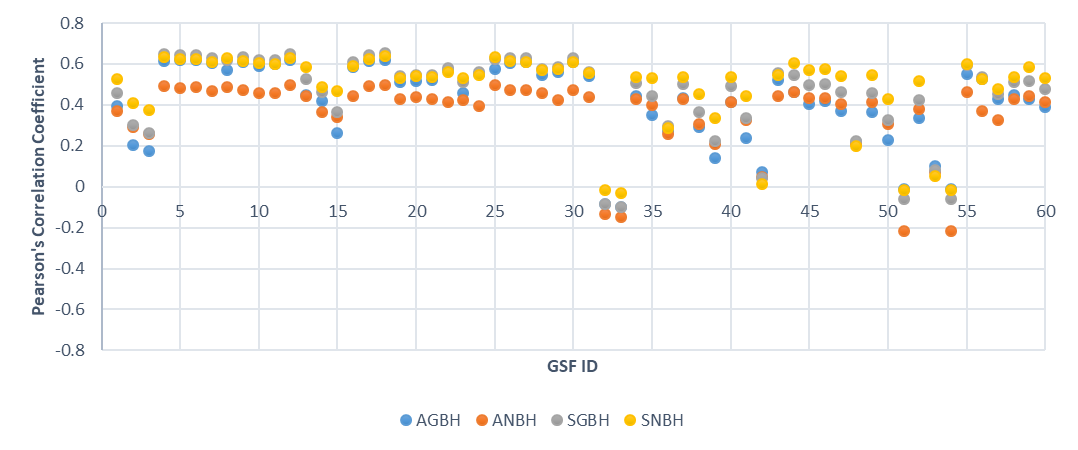


DEM: MERIT


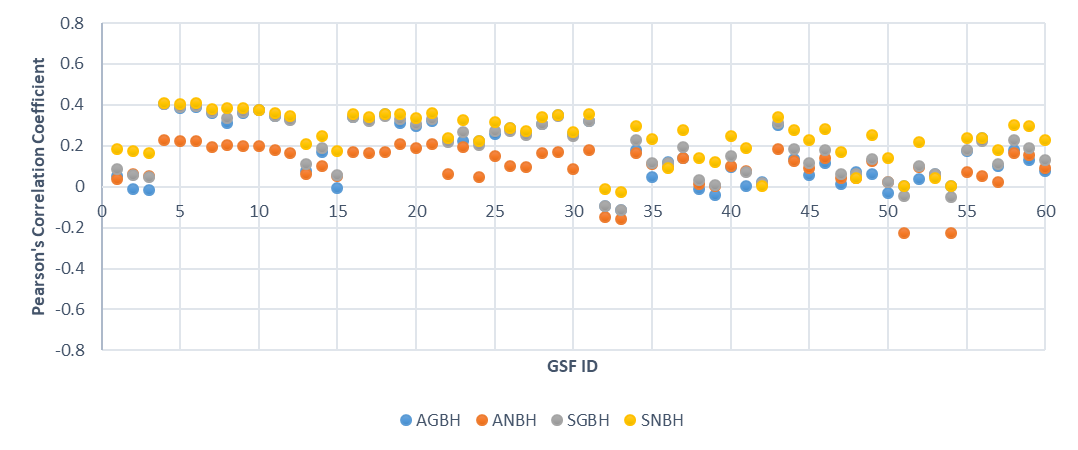


DEM: ALOS


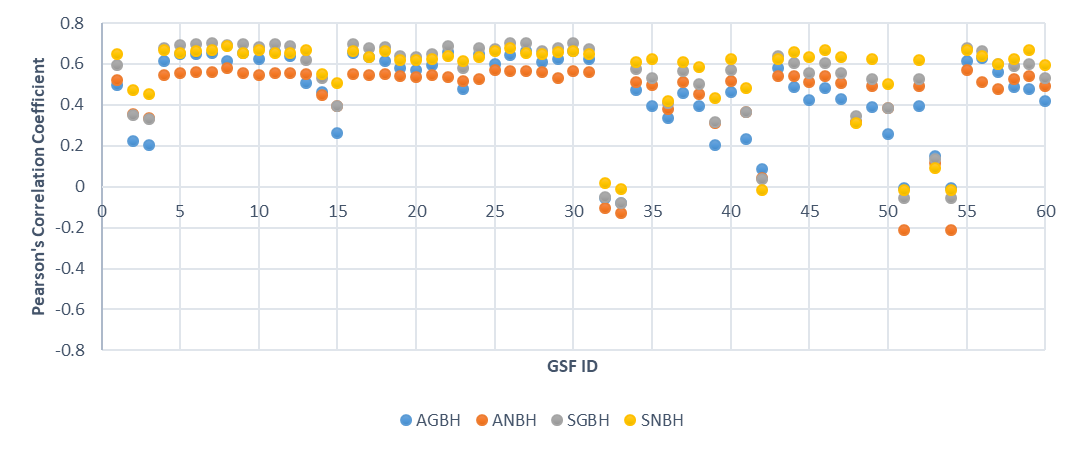


DEM: ASTER

DEM: AW3D30


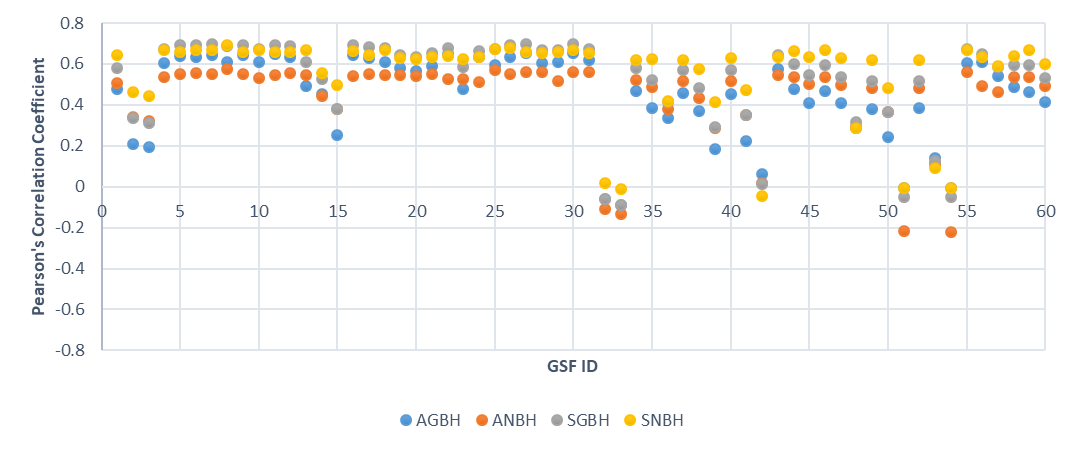


DEM: CMP_SRTM30-AW3D30_U


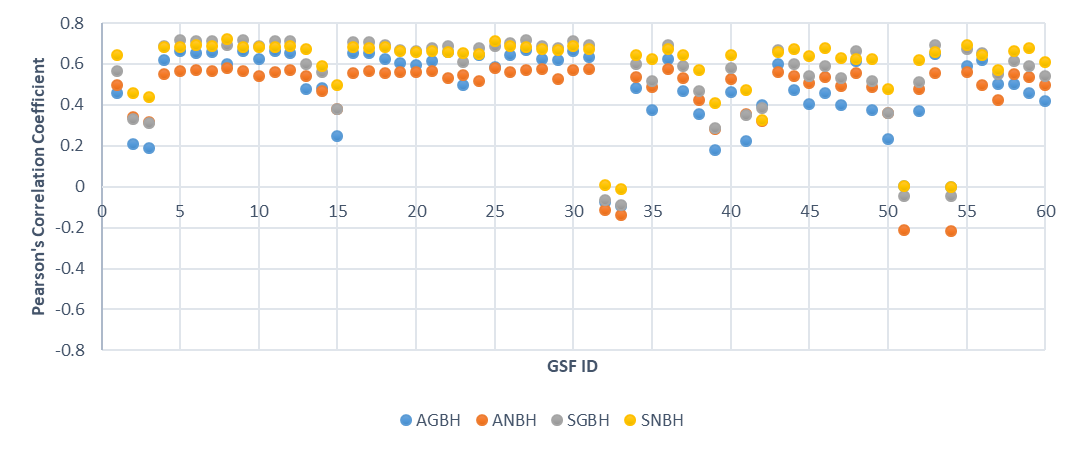


DEM: CMP_SRTM30-AW3D30_I


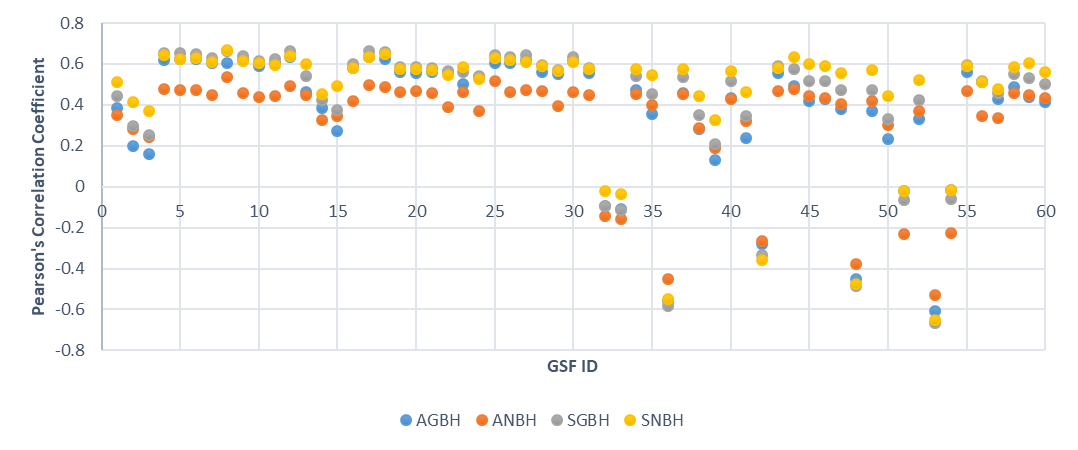


# References

[1] B. Rabus, M. Eineder, A. Roth, and R. Bamler, “The shuttle radar topography mission—a new class of digital elevation models acquired by spaceborne radar,” *ISPRS Journal of Photogrammetry and Remote Sensing*, vol. 57, no. 4, pp. 241–262, Feb. 2003, doi: 10.1016/S0924-2716(02)00124-7.

[2] M. Mukul, V. Srivastava, and M. Mukul, “Accuracy analysis of the 2014–2015 Global Shuttle Radar Topography Mission (SRTM) 1 arc-sec C-Band height model using International Global Navigation Satellite System Service (IGS) Network,” *Journal of Earth System Science*, vol. 125, no. 5, pp. 909–917, Jul. 2016, doi: 10.1007/s12040-016-0716-8.

[3] J. R. Santillan and M. Makinano-Santillan, “Vertical Accuracy Assessment of 30-M Resolution Alos, Aster, and Srtm Global Dems Over Northeastern Mindanao, Philippines,” *ISPRS - International Archives of the Photogrammetry, Remote Sensing and Spatial Information Sciences*, pp. 149–156, Jun. 2016, doi: 10.5194/isprs-archives-XLI-B4-149-2016.

[4] USGS Earth Explorer, *U.S. Geological Survey*. .

[5] T. Tachikawa, M. Hato, M. Kaku, and A. Iwasaki, “Characteristics of ASTER GDEM version 2,” in *2011 IEEE international geoscience and remote sensing symposium*, 2011, pp. 3657–3660.

[6] C. Jing, A. Shortridge, S. Lin, and J. Wu, “Comparison and validation of SRTM and ASTER GDEM for a subtropical landscape in Southeastern China,” *International Journal of Digital Earth*, vol. 7, no. 12, pp. 969–992, Nov. 2014, doi: 10.1080/17538947.2013.807307.

[7] I. Elkhrachy, “Vertical accuracy assessment for SRTM and ASTER Digital Elevation Models: A case study of Najran city, Saudi Arabia,” *Ain Shams Engineering Journal*, vol. 9, no. 4, pp. 1807–1817, Dec. 2018, doi: 10.1016/j.asej.2017.01.007.

[8] T. Tadono *et al.*, “Generation of the 30 M-Mesh Global Digital Surface Model by Alos Prism,” *ISPRS - International Archives of the Photogrammetry, Remote Sensing and Spatial Information Sciences*, pp. 157–162, Jun. 2016, doi: 10.5194/isprs-archives-XLI-B4-157-2016.

[9] D. Yamazaki *et al.*, “A high-accuracy map of global terrain elevations,” *Geophysical Research Letters*, vol. 44, no. 11, pp. 5844–5853, May 2017, doi: 10.1002/2017GL072874.

[10] J. Serra, *Image analysis and mathematical morphology*. Academic Press, Inc., 1983.

[11] M. Pesaresi and J. A. Benediktsson, “A new approach for the morphological segmentation of high-resolution satellite imagery,” *Geoscience and Remote Sensing, IEEE Transactions on*, vol. 39, no. 2, pp. 309–320, Feb. 2001, doi: 10.1109/36.905239.

[12] R. M. Haralick, K. Shanmugam, and others, “Textural features for image classification,” *IEEE Transactions on systems, man, and cybernetics*, no. 6, pp. 610–621, 1973.

[13] M. Pesaresi, A. Gerhardinger, and F. Kayitakire, “A Robust Built-Up Area Presence Index by Anisotropic Rotation-Invariant Textural Measure,” *IEEE Journal of Selected Topics in Applied Earth Observations and Remote Sensing*, vol. 1, no. 3, pp. 180–192, Sep. 2008, doi: 10.1109/JSTARS.2008.2002869.
